# The hijacking of a wheat *Exo70*-like protein by a fungal pathogen triggers disease

**DOI:** 10.1038/s41467-026-77720-9

**Published:** 2026-09-10

**Authors:** Gurminder Singh, Katherine L. D. Running, Zengcui Zhang, Sudeshi Seneviratne, Krishna Acharya, Tiana M. Roth, Amanda R. Peters Haugrud, Agnes Szabo-Hever, Pooja Kumari, Phillip E. McClean, Zhaohui Liu, Shengming Yang, Justin D. Faris

**Affiliations:** 1 Department of Plant Sciences, North Dakota State University, Fargo, ND USA; 2 Department of Plant Pathology, North Dakota State University, Fargo, ND USA; 3 USDA-ARS, Edward T. Schafer Agricultural Research Center, Fargo, ND USA; 4 Deceased: Phillip E. McClean, Fargo, ND USA

**Keywords:** Plant genetics, Plant immunity, Plant breeding

## Abstract

Tan spot is an economically important fungal disease of wheat that occurs globally. In this necrotrophic pathosystem, pathogen effectors are recognized by wheat genes in an inverse gene-for-gene manner, leading to the hijacking of host defense mechanisms and ultimately disease susceptibility. Here, we identified the tan spot susceptibility gene *Tsc2* through positional cloning, mutagenesis, and transgenic complementation. *Tsc2* encodes a diverged form of the exocyst subunit Exo70FX15 that localizes to the nucleus and cytoplasm and does not interact directly with the fungal effector. A 21‑nucleotide deletion in *Tsc2* occurred in durum wheat more than a century ago and subsequently spread to common wheat through breeding practices, which allowed recognition of the pathogen effector and subsequent hijacking of the host immune system. We demonstrate that elimination of *Tsc2* through conventional breeding using a diagnostic marker or disruption of *Tsc2* by gene editing can be used to improve tan spot resistance in wheat.

## Abstract (web summary)

Tan spot is an economically important disease of wheat. Here the authors show that a deletion in the wheat gene *Tsc2*, which encodes an Exo70-like protein, enables a fungal effector to hijack host immunity causing cell death and demonstrate practical routes to tan spot resistance.

## Introduction

Wheat (*Triticum* spp.) is cultivated on more than 215 million hectares and supplies nearly 20% of the calories and protein in the human diet worldwide^1^. As global demand for wheat continues to rise, safeguarding its productivity has become a critical priority for food security^2^. Yet several foliar diseases continue to erode attainable yields. Among these, tan spot (also known as yellow spot), caused by the necrotrophic fungus *Pyrenophora tritici-repentis* (Ptr), has emerged as a globally pervasive and damaging foliar disease of common (bread) wheat (*T. aestivum* L., 2*n* = 6*x* = 42, AABBDD genomes) and durum (pasta) wheat (*T. turgidum* L. ssp. *durum* (Desf.) van Slageren, 2*n* = 4*x* = 28, AABB genomes) that compromises both grain yield and quality^3–5^. Recent global burden analyses rank tan spot among the six most damaging wheat diseases^6^, costing growers hundreds of millions of dollars and resulting in losses of nearly 22.4 billion loaves of bread annually^7^. Meta-analyses estimate average yield losses of 5–10%, with documented epidemic losses of up to 50% under conducive conditions^6,8–11^. The rapid expansion of conservation tillage that began in the 1970s has intensified disease pressure by allowing Ptr to overwinter on infected crop residue, and warming weather patterns are expected to extend the pathogen’s geographic range^5,12,13^.

Current management of tan spot relies on agronomic practices such as crop rotation, residue management, and timely fungicide applications to reduce inoculum and suppress disease development^14–16^. However, these approaches are constrained by agronomic feasibility, economic cost, and environmental sustainability. Genetic resistance offers the most durable and environmentally sound solution, but breeding progress has been hampered by the distinctive pathogenic strategy of Ptr^5^.

Biotrophs such as the rust and mildew pathogens require living tissue to acquire nutrients and are typically thwarted when classical resistance (R) genes trigger programmed cell death (PCD) and associated defense responses. But contrary to biotrophic pathogens, necrotrophic pathogens require dead or dying cells to acquire nutrients. To achieve this, Ptr employs necrotrophic effectors (NEs) that act to hijack the plant’s immune system through recognition by specific host genes that activate PCD and the ‘defense response’^5,17^. Therefore, the lifestyles of necrotrophs such as Ptr have made it possible for them to obtain the ability to exploit the innate immune systems of plants for their own benefit.

Three Ptr-produced NEs named ToxA, ToxB, and ToxC have been characterized^18–20^. These NEs rely on recognition by the dominant host genes *Tsn1*, *Tsc2*, and *Tsc1*, respectively, to trigger susceptibility^5^. This inverse gene-for-gene system has become a foundational model for understanding necrotrophic plant-pathogen interactions^21^.

The tan spot susceptibility genes *Tsn1* and *Tsc1* have been cloned and functionally characterized. *Tsn1* encodes a protein with integrated serine/threonine protein kinase (PK), nucleotide-binding, and leucine-rich repeat (NLR) domains^17^. *Tsc1* encodes a membrane-spanning leucine-rich repeat receptor-like kinase (LRR-RLK)^22,23^. The identification and characterization of *Tsn1* and *Tsc1* have demonstrated that Ptr can subvert immune-receptor architecture involving both effector- and pathogen-associated molecular pattern (PAMP)-triggered immunity (ETI and PTI) pathways to drive host cell death^21^.

The role of a compatible *Tsc2*-ToxB interaction in tan spot development has been well established in both common wheat^24–27^ and durum wheat^28–30^. ToxB is a 6.5-kDa, cysteine-rich secreted effector encoded by a gene that exists in multiple tandem copies on a mobile 294-kb “Starship” element^31^. Population surveys revealed that this locus undergoes frequent duplication, loss, and diversifying selection, shaping virulence diversity worldwide^32,33^. Fluorescent tagging, apoplastic fluid extractions, and heterologous expression experiments demonstrated that ToxB remains extracellular, and it alone is sufficient to induce chlorosis in the presence of *Tsc2*, indicating that it acts in the leaf apoplast rather than entering host cells^34,35^. Previous linkage studies placed *Tsc2* on wheat chromosome arm 2BS and delimited it to a 1.9 Mb region, but the causal gene has not been identified^24,26,27,30^.

Here, we show that *Tsc2* encodes a previously uncharacterized member of the exocyst subunit Exo70 family that is necessary and sufficient to confer ToxB-triggered cell death. Natural or induced loss-of-function alleles abolish effector sensitivity and confer resistance to tan spot. A 21-nucleotide (nt) deletion in *Tsc2* that likely occurred in a domesticated durum wheat variety and subsequently spread to hexaploid common wheat through breeding practices is required for ToxB recognition and the subsequent hijacking of defense mechanisms. Together, these findings deepen our understanding of the functionalization of *Exo70*-like genes beyond conferring resistance to biotrophic pathogens to serving as susceptibility targets for necrotrophic pathogens. Finally, the outcomes of this research deliver practical tools such as diagnostic markers and gene-editing methods to support the development of more resilient cultivars.

## Results

### *Tsc2* mapping and candidate gene identification

**Fig. 1:**
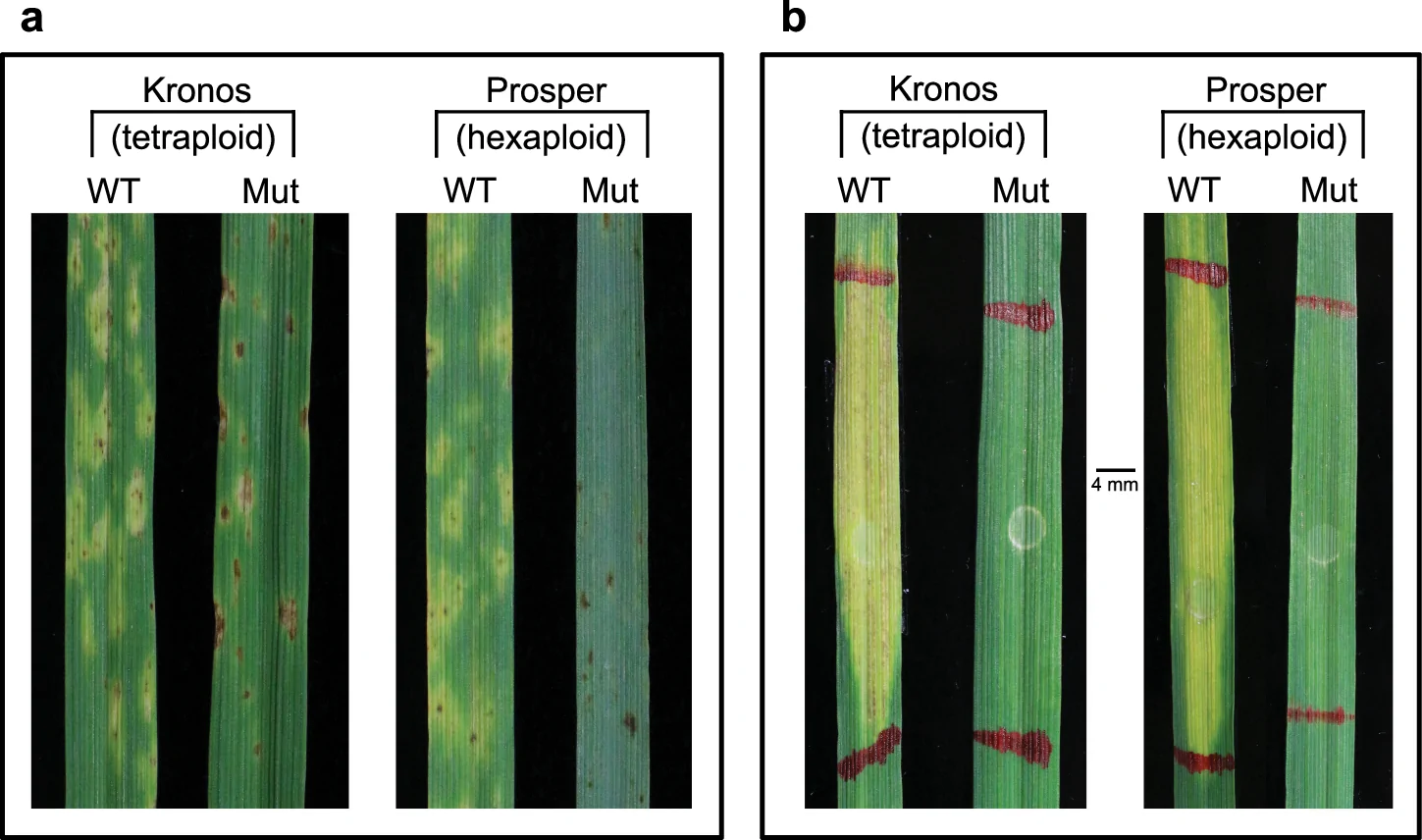
Phenotypic effects of a compatible *Tsc2*-ToxB interaction in tetraploid (durum) and hexaploid (common) wheat. **a** Spray inoculation with the ToxB-producing *Pyrenophora tritici-repentis* race 5 isolate DW5. Wild-type durum wheat cultivar Kronos (tetraploid) and common wheat cultivar Prosper (hexaploid), which carry a functional *Tsc2* allele, exhibit expanding and coalescing chlorotic lesions in response to infection, whereas their corresponding *Tsc2-*disrupted mutants (Kronos2704 and Prosper89a) show infection site lesions with little to no chlorosis development or expansion. **b** Leaf infiltration of cultures containing the necrotrophic effector ToxB. Wild-type Kronos and Prosper develop localized cell death at infiltration sites, whereas the *Tsc2-*disrupted mutants undergo no cell death. WT wild type; Mut *Tsc2-*disrupted mutant. The images in (**a**) and (**b**) are representative of three independent spray-inoculation experiments and three independent ToxB-infiltration experiments, respectively; all experiments produced similar results.

**Fig. 2:**
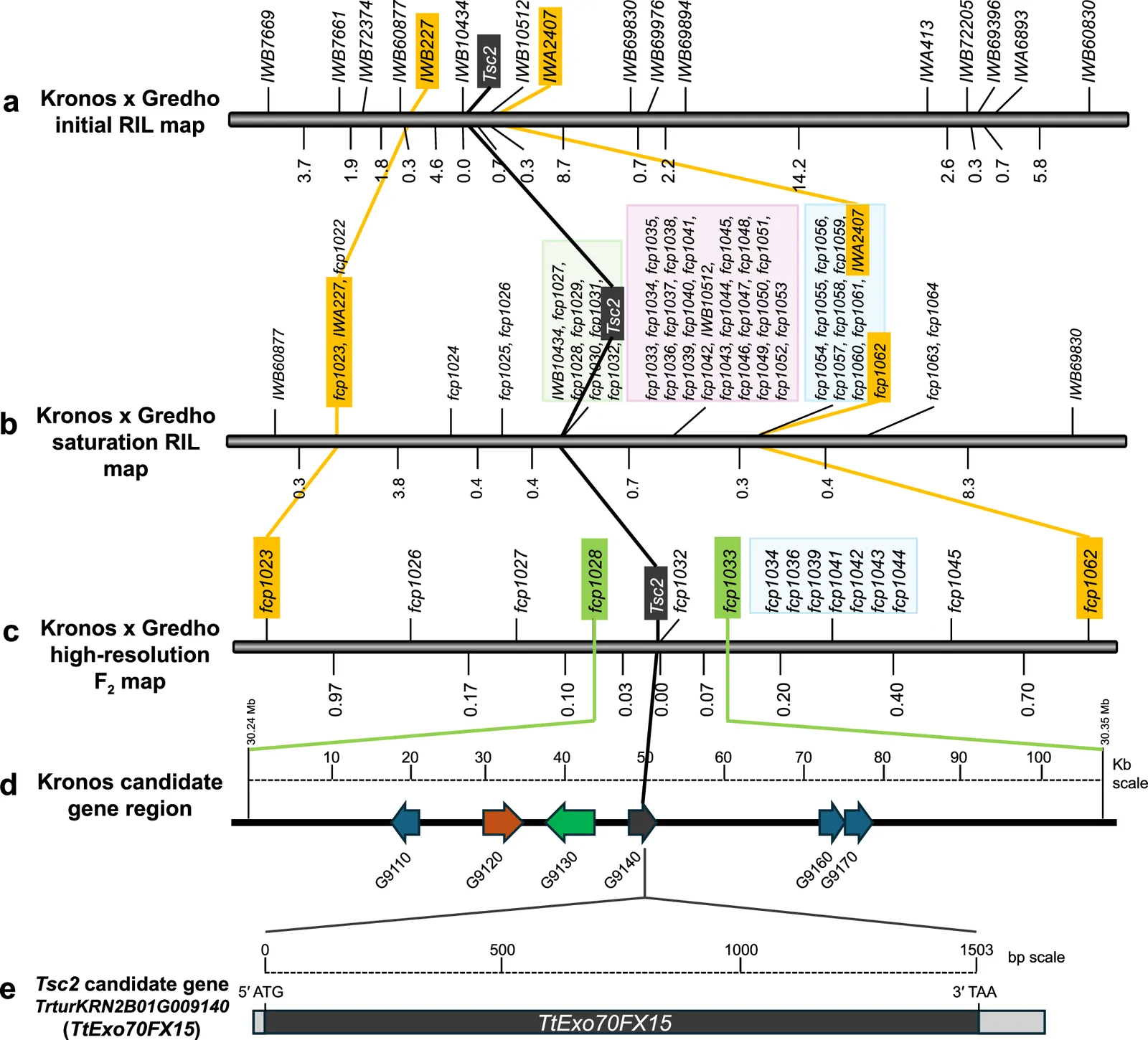
Mapping and identification of candidate genes for *Tsc2*. **a** The initial genetic linkage map of the *Tsc2* region of chromosome arm 2BS generated in the Kronos × Gredho recombinant inbred population. The flanking markers used to define the target interval for marker development are highlighted in yellow. **b** Saturated linkage map developed in the Kronos × Gredho recombinant inbred population by developing 43 additional markers. **c** High-resolution linkage map developed in the Kronos × Gredho F_2_ population between markers *fcp1023* and *fcp1062*. Along the genetic maps of (**a**), (**b**), and (**c**), marker names are shown above the gray bars, and genetic distances in centimorgans (cM) are shown below. **d** Physical map of the 108.4 kb candidate interval between markers *fcp1028* and *fcp1033* in the Kronos genome. Arrows indicate predicted gene models and are colored according to predicted function: blue, transferase-family genes (*TrturKRN2B01G009110*, *TrturKRN2B01G009160*, and *TrturKRN2B01G009170*); red, protein containing an uncharacterized plant invertase/pectin methylesterase inhibitor-like domain (*TrturKRN2B01G009120*); green, leucine-rich repeat receptor-like kinase (LRR-RLK; *TrturKRN2B01G009130*); and dark gray, Exo70 exocyst subunit-family gene subsequently identified as *Tsc2*/*TtExo70FX15* (*TrturKRN2B01G009140*). **e** The structure of candidate gene *TrturKRN2B01G009140* (*TtExo70FX15*), which consists of a single exon (dark gray). The untranslated regions are indicated by light gray shaded boxes.

The *ToxB* gene was cloned into a yeast expression vector of *Pichia pastoris*^36^, and cultures of the recombinant *P. pastoris* expressing the ToxB protein were infiltrated into wheat leaves to determine if plants were sensitive or insensitive as a proxy for ToxB-induced disease^30,34^ (Fig. 1). Plants that display cell death in the form of chlorosis carry the dominant *Tsc2* allele, whereas those showing no reaction have a recessive or absent allele^5^. A tetraploid wheat mapping population of 149 recombinant inbred lines (RILs) derived from a cross between the durum cultivars Kronos (ToxB-sensitive) and Gredho (ToxB-insensitive) (KG RIL population) was genotyped with the Illumina iSelect 90 K SNP array (Supplementary Data 1) and infiltrated with ToxB cultures (Supplementary Data 2). Genetic linkage analysis delineated the *Tsc2* locus to a 5.6 cM interval on the short arm of chromosome 2B (Fig. 2a), consistent with its previously reported position^24,26,27,30^.

To saturate the *Tsc2* region between SNP markers *IWB227* and *IWA2407*, we developed 43 additional simple sequence repeat (SSR) and Kompetitive allele‑specific PCR (KASP) markers (*fcp1022* to *fcp1064*) based on the intervening genome sequence in Kronos^37^ (Fig. 2b, Supplementary Data 3). In the revised chromosome 2B genetic map, these markers further delineated the *Tsc2* locus to 1.1 cM.

To further refine the region, 7002 F_2_ plants from Kronos × Gredho were infiltrated with ToxB, and 1492 insensitive plants (homozygous recessive for *Tsc2*) were used for high-resolution mapping. Among these, 159 plants with recombination events between markers *fcp1023* and *fcp1062* were identified. Genotyping of these recombinants further delimited *Tsc2* to a 0.1 cM interval flanked by markers *fcp1028* and *fcp1033* (Fig. 2c). Alignment with the Kronos genome assembly^37^ placed this segment at 30.240–30.349 Mb on chromosome 2B, a span of 108.4 kb harboring six predicted gene models including three transferase-family genes, one gene encoding an uncharacterized plant invertase/pectin methylesterase inhibitor-like domain protein, one LRR-RLK, and one Exo70 exocyst subunit-family gene (Fig. 2d, Supplementary Data 4).

The gene content of the 108.4 kb interval was examined in 12 wheat lines with available sequenced genomes, which included four ToxB-sensitive lines and eight insensitive lines (Supplementary Data 5). Four of the six genes were conserved across all 12 lines, whereas two genes displayed polymorphisms between sensitive and insensitive lines and were considered *Tsc2* candidates. One candidate gene, *TrturKRN2B01G009130*, encoded an LRR-RLK and was intact in the genomes of the four ToxB-sensitive lines, but it was either absent or carried nonsynonymous SNPs in the insensitive lines. The second candidate gene, *TrturKRN2B01G009140* (Fig. 2e), encoded an unconventional Exo70 subunit (hereafter referred to as *TtExo70FX15*). This gene was 100% identical in all ToxB-sensitive lines, and it was either absent or lacking a 21-nt in-frame deletion in all insensitive lines.

### Validation of *Tsc2* candidates by loss-of-function mutants

To determine if either candidate gene was necessary for ToxB sensitivity, we evaluated Kronos Targeting Local Lesions IN Genomes (TILLING) lines^38^ that carried nonsense or predicted deleterious missense mutations in either the LRR-RLK gene (*TrturKRN2B01G009130*) or *TtExo70FX15*. Of 19 independent TILLING lines with mutations in the LRR-RLK gene, all plants exhibited cell death following infiltration, the same as wild-type Kronos, demonstrating that *TrturKRN2B01G009130* was not necessary for ToxB sensitivity (Supplementary Data 6).

**Fig. 3:**
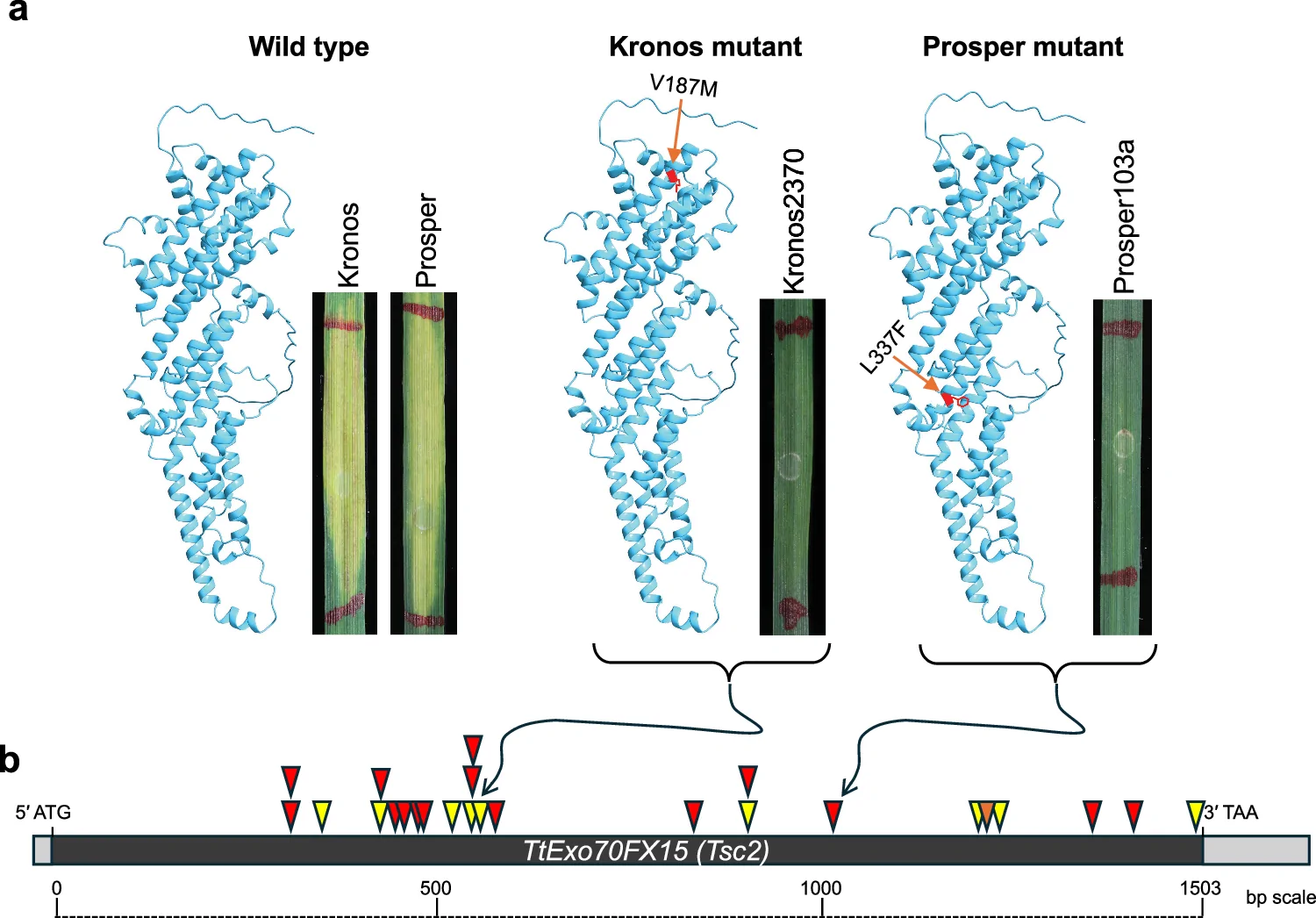
Evaluation and analysis of mutations in the *TtExo70FX15* (*Tsc2*) gene in Kronos and Prosper. **a** ToxB-infiltration reactions and predicted TtExo70FX15 (Tsc2) protein structures of the wild-type wheat lines Kronos and Prosper, and one representative missense mutant in each of the two lines that results in an insensitive reaction to ToxB. Protein structures depict the AlphaFold-predicted wild-type TtExo70FX15 (Tsc2) protein, with missense mutation sites highlighted to indicate their spatial positions within the structure. The locations of the point mutations in the two mutant proteins are indicated in red and indicated by orange arrows. Infiltration reactions and predicted protein structures for all other Kronos and Prosper *TtExo70FX15* (*Tsc2*) missense mutants are presented in Supplementary Figs. 1 and 2. **b** The relative locations of all mutations evaluated are indicated by colored arrows. Red arrows indicate locations of mutations in Prosper. Yellow and orange indicate locations of mutations in Kronos, with yellow denoting mutations that confer ToxB insensitivity and the single orange mutation resulting in a sensitive reaction (i.e., no change from wild type). Black arrows indicate the physical positions of the two mutations shown in (**a**) along the gene structure.

Ten Kronos TILLING lines with nonsense or missense mutations in *TtExo70FX15* were examined (Supplementary Data 7). Among those, four with nonsense mutations (W149*, W301*, W391*, and Q395*) and five with missense mutations (E125V, A175T, A186T, V187M, and G500E) were insensitive to ToxB (Fig. 3, Supplementary Fig. 1). One missense variant (H393Y) in TILLING line Kronos3105 with tolerated SIFT scores and neutral Missense3D predictions retained sensitivity. The retention of sensitivity in the H393Y mutant may reflect its predicted stabilizing effect (Dynamut2 ΔΔG = +1.13 kcal/mol), which could maintain native protein conformation and function.

Independent corroboration came by conducting a forward genetic screen of 1050 ethyl methanesulfonate (EMS)-treated M_2_ families of the ToxB-sensitive hexaploid wheat cultivar Prosper. Fifteen insensitive M_2_ families were recovered, and comparative sequence analysis revealed that each family carried a nonsense or missense mutation in the Prosper copy of *TtExo70FX15* (Supplementary Data 7, Fig. 3, Supplementary Fig. 2). Taken together, every protein-truncating mutation and all but one missense mutation (Kronos3105) in *TtExo70FX15* converted ToxB-sensitive wheat to an insensitive state. In contrast, none of the mutations in the LRR-RLK gene (*TrturKRN2B01G009130*) affected ToxB sensitivity. These results confirmed that *TtExo70FX15* is necessary for ToxB sensitivity, and together with the results of genetic linkage analysis, provided strong evidence that it was *Tsc2*.

### Transgenic complementation confirms *TtExo70FX15* as *Tsc2*

**Fig. 4:**
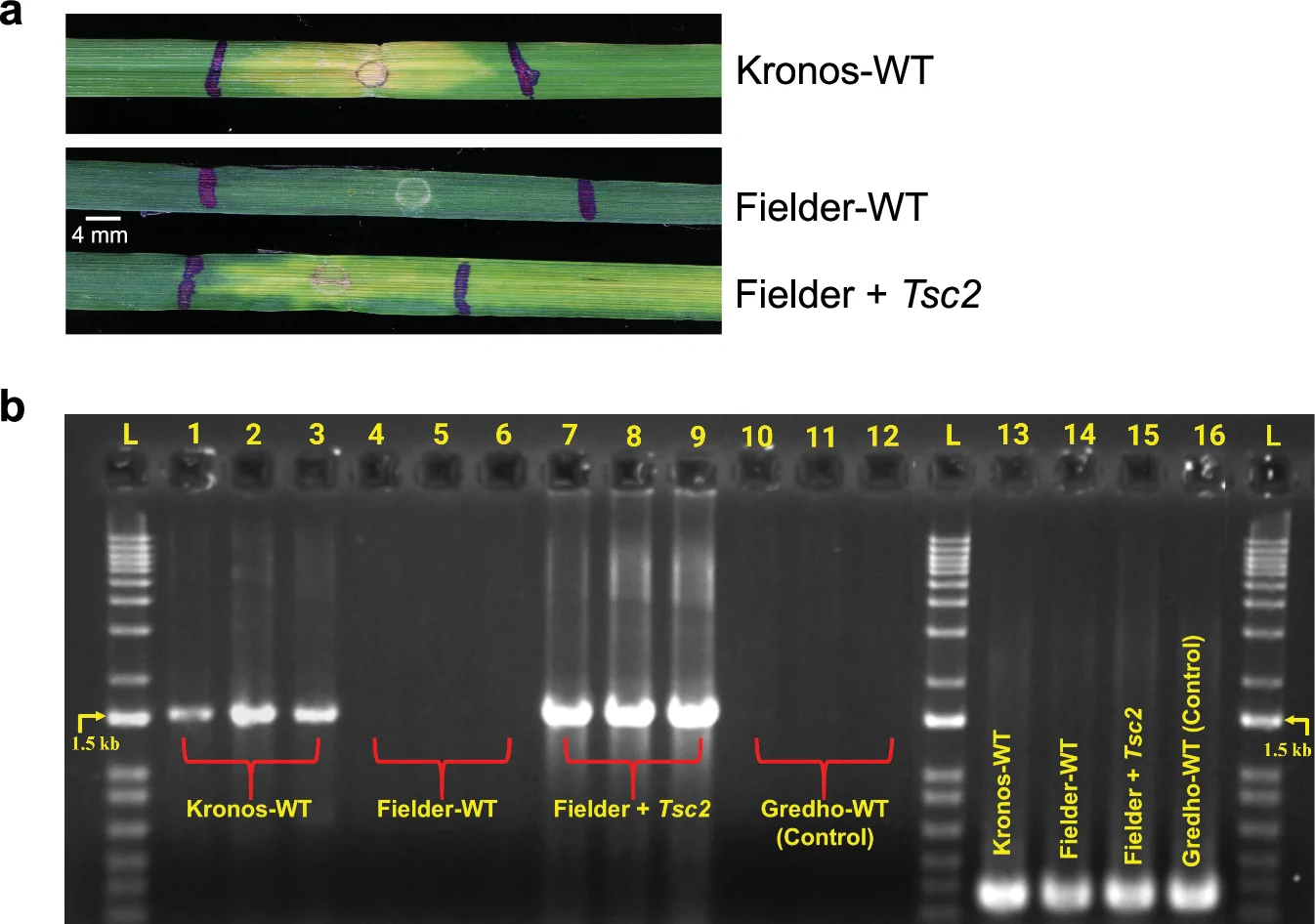
Genetic complementation by transformation of the wheat cultivar Fielder with the *TtExo70FX15* (*Tsc2*) gene. **a** ToxB infiltration of leaves of the durum cultivar Kronos, the common wheat cultivar Fielder, and Fielder transformed with the Kronos *Tsc2* (*TtExo70FX15)* gene, including ~2 kb upstream and ~2 kb downstream of the native start and stop codons (Fielder + *Tsc2*). **b** Agarose gel electrophoresis of PCR-amplified fragments representing the 1.5 kb *TtExo70FX15* open reading frame in Kronos, Fielder, transgenic Fielder + *TtExo70FX15* (Fielder *+ Tsc2*), and Gredho (ToxB-insensitive and negative control) (lanes 1–12). The PCR experiments were done in triplicate. Lanes 13–16 show the PCR amplification of the endogenous *GAPDH* gene as a positive PCR control for each DNA sample. Source data are provided as a Source Data file.

To determine whether *TtExo70FX15* is sufficient to confer sensitivity to ToxB, a 6.5-kb genomic fragment comprising the Kronos *TtExo70FX15* coding sequence plus its native promoter and terminator was introduced into the ToxB-insensitive hexaploid spring wheat cultivar Fielder. A single T_0_ transformant was verified by gene-specific PCR and Sanger sequencing. Infiltration of leaves with ToxB revealed that all transgene-positive tillers were as sensitive as Kronos (Fig. 4). In contrast, wild-type Fielder and Gredho controls remained symptom-free. The gain of sensitivity in Fielder, together with loss of insensitivity in nine independent Kronos TILLING lines and 15 independent Prosper EMS lines, demonstrated that *TtExo70FX15* was both necessary and sufficient for ToxB-induced cell death. Therefore, *TtExo70FX15* was demonstrated to be the causal gene functioning as *Tsc2*, and it will hereafter be referred to as the latter.

### *Tsc2* characterization and transcriptional expression

**Fig. 5:**
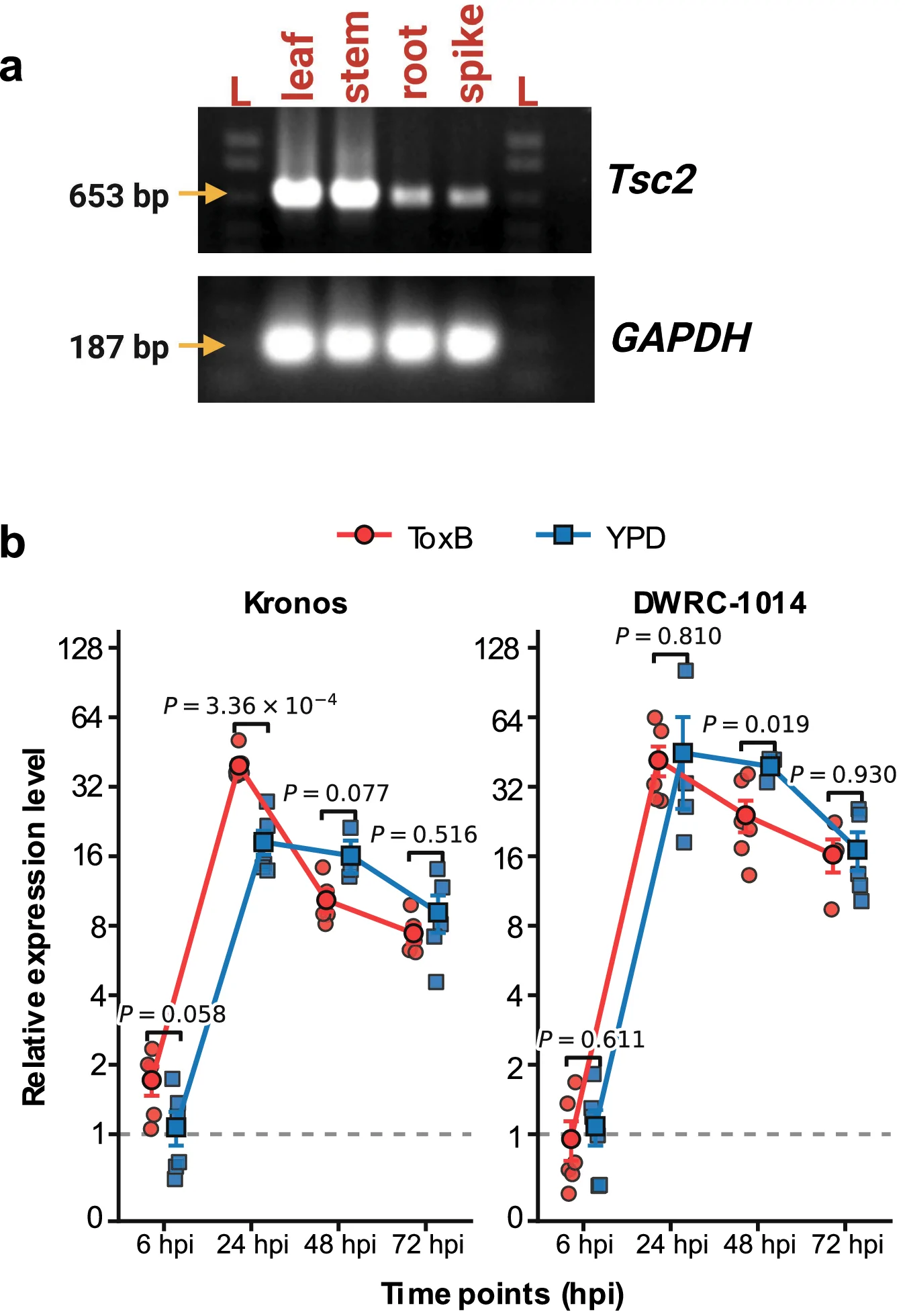
Transcriptional expression of *Tsc2*. **a** Transcriptional expression analysis of *Tsc2* in different wheat tissues using reverse transcription-PCR (RT-PCR). Amplification of the housekeeping gene *GAPDH* served as an endogenous control; the observed *GAPDH* amplicon corresponds to the expected cDNA product (187 bp), and no genomic DNA-derived product (357 bp) was detected. **b** Temporal expression dynamics of *Tsc2* in two-week-old Kronos (ToxB-sensitive; left) and DWRC-1014 (ToxB-insensitive; right) seedlings following infiltration with ToxB-producing culture (ToxB) and control yeast-peptone-dextrose (YPD) culture medium not producing ToxB. Quantitative real-time (qRT)-PCR was performed at 6, 24, 48, and 72 h post-infiltration (hpi). *Tsc2* expression was normalized to *GAPDH*, and relative expression was calculated using the 2^-ΔΔCq^ method. The y-axis is displayed on a log_2_ scale, and the dashed horizontal line indicates a relative expression value of 1. Lines connect arithmetic means; error bars represent the standard error of the mean (SEM); jittered dots represent individual biological replicates. Statistical differences between treatments (ToxB and YPD) were assessed by two-sided Welch’s *t*-tests; exact *P*-values are shown above each comparison. Source data are provided as a Source Data file. Detailed Cq values and statistical summaries are provided in Supplementary Data 8 and 9.

cDNA sequencing revealed *Tsc2* is an intronless gene of 1503 bp (Fig. 2E) that encodes a 500-amino-acid protein. The sizes of the untranslated regions (UTRs) were variable, with the 5′ UTR ranging from 13 to 153 bp and the 3′ UTR from 132 to 244 bp, but sizes of 19 and 145 bp occurred most frequently for the 5′ and 3′ UTRs, respectively. Reverse transcription (RT)-PCR detected abundant *Tsc2* transcripts in leaf and stem tissues, but comparatively less in root and immature spike tissue (Fig. 5).

To determine whether *Tsc2* transcription is modulated by ToxB, leaves of two-week-old Kronos plants (ToxB-sensitive; Haplotype 1) and DWRC-1014 (ToxB-insensitive; Haplotype 3) were infiltrated with ToxB culture or with a control culture medium [yeast-peptone-dextrose (YPD)] not producing ToxB. Samples were collected at 6, 24, 48, and 72 h post-infiltration (hpi), and *Tsc2* mRNA abundance was quantified by quantitative real-time (qRT)-PCR relative to *GAPDH* (Supplementary Data 8; Fig. 5; Supplementary Data 9). In Kronos, *Tsc2* transcript levels increased significantly more in the ToxB-treated leaves compared to YPD-treated leaves within the first 24 h. Transcript levels then declined to similar levels at 48 and 72 hpi in both ToxB- and YPD-treated leaves. A nearly identical pattern of transcription was observed in the ToxB-insensitive line DWRC-1014 except that *Tsc2* transcription was not significantly greater in ToxB-treated leaves compared to YPD-treated leaves at 24 hpi. This indicates that ToxB upregulates *Tsc2* transcription when recognized in a compatible interaction, which likely leads to the rapid induction of PCD and the defense response.

### Phylogenetic analysis, domain architecture, and subcellular localization of Tsc2

**Fig. 6:**
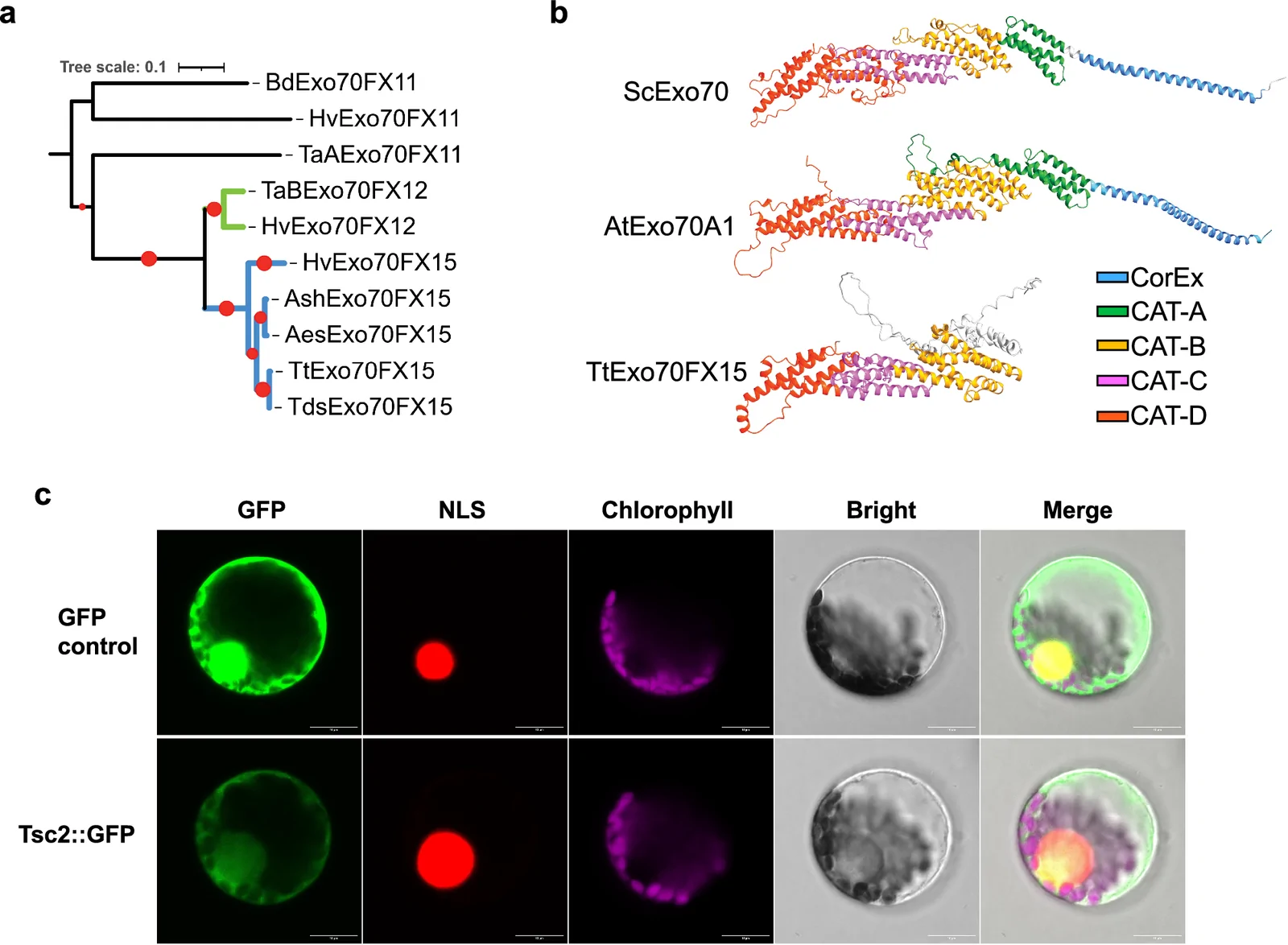
Phylogenetics, structure, and localization of the Tsc2 protein. **a** Rooted maximum-likelihood (ML) phylogenetic tree of the Tsc2 protein relative to other Exo70FX proteins of the Poales, reconstructed from previously described datasets^40^ by adding Exo70FX15 from *Aegilops* and *Triticum* species. Protein sequences were aligned using structure-guided multiple sequence alignment, and the tree was inferred using RAxML with a GAMMA model of rate heterogeneity. Red circles indicate bootstrap support greater than 80%. Branch lengths are proportional to the number of amino acid substitutions per site; the scale bar indicates 0.1 substitutions per site. Bd *Brachypodium distachyon*, Hv *Hordeum vulgare* (barley), Ta *Triticum aestivum* (common wheat), Ash *Aegilops sharonensis*, Aes *Aegilops speltoides*, Tt *Triticum turgidum* ssp. *durum*, Tds *Triticum turgidum* ssp. *dicoccoides*. **b** Predicted 3D structure of the wheat TtExo70FX15 (Tsc2) protein compared to crystallized structures of yeast (ScExo70; PDB 2B1E) and Arabidopsis (AtExo70A1; PDB 4RL5) proteins, demonstrating the absence of the CorEX and CAT-A domains in TtExo70FX15 (Tsc2). **c** Subcellular localization of TtExo70FX15 (Tsc2) and green fluorescent protein (GFP) control. Confocal microscopy images show fluorescence signals in the GFP channel (green), a nuclear localization signal (NLS) protein fused to mKATE, chlorophyll autofluorescence channel (red), bright-field channel, and merged images. Scale bar = 10 μm. Two independent wheat protoplast localization experiments were performed and produced similar localization patterns. Representative images from the second experiment, which included the NLS-mKATE nuclear marker, are shown. Source data are provided as a Source Data file.

The plant exocyst is an octameric vesicle-tethering complex composed of Sec3, Sec5, Sec6, Sec8, Sec10, Sec15, Exo70, and Exo84 subunits^39^. During land plant evolution, Exo70 expanded into ten clades (A, B, C, D, E, F, G, H, I, and J), while a further, highly divergent clade, Exo70FX, arose after the Poales order diverged from other monocots and subsequently radiated in the grasses^40^. To establish the evolutionary relationship of *Tsc2*, we appended its amino acid sequence to the FX11/FX12/FX15 data sets reported previously^40^ and rebuilt a structure-guided, maximum-likelihood phylogeny (Fig. 6). Tsc2 grouped decisively with the FX15 branch, indicating it is an Exo70FX15 isoform. Searches of representative non-Triticeae grasses, including rice, maize, and sorghum, revealed divergent Exo70FX-family members but no orthologs of the Triticeae Exo70FX15/Tsc2. Thus, Tsc2 belongs to a Triticeae-specific Exo70FX15 lineage that is distinct from the broader Exo70FX diversification observed across Poales.

We next examined whether the Tsc2 protein preserves the architectural signatures of canonical Exo70s. AlphaFold2 modeling, followed by superimposition onto yeast (PDB: 2B1E) and *Arabidopsis* (PDB: 4RL5) Exo70 structures, showed that Tsc2 retains the canonical CAT-B, CAT-C, and CAT-D domains but lacks both the N-terminal CorEx helix and the CAT-A domain (Fig. 6). CorEx forms the four-helix zipper that docks Exo70 onto Exo84 and nucleates exocyst assembly. CAT-A, together with CAT-D, contributes to phospholipid binding at the plasma membrane^39,41^. Loss of both elements suggests that Tsc2 is unlikely to participate in a canonical exocyst and has instead acquired a specialized function.

Consistent with its predicted divergence from canonical Exo70 proteins, Tsc2-GFP exhibited a nucleocytoplasmic distribution when transiently expressed in wheat mesophyll protoplasts together with the nuclear-marker NLS-mKate2 (Fig. 6c). GFP fluorescence was detected within the NLS-mKate2-positive nuclear compartment as well as in the surrounding cytoplasm, indicating that Tsc2 is present in both compartments. Fluorescence intensity profiles across five independent transformed protoplasts confirmed overlap between Tsc2-GFP signal and the nuclear-marker-defined region, while also showing signal outside the nucleus (Supplementary Fig. 4). Under these assay conditions, Tsc2-GFP did not display the discrete plasma membrane-enriched pattern expected for canonical Exo70 subunits, supporting the notion that Tsc2 has neofunctionalized and acts outside the canonical vesicle-tethering pathway.

To determine if the Tsc2 protein directly interacts with ToxB, we conducted a yeast two-hybrid (Y2H) assay using ToxB as bait and full-length Tsc2 as prey in the Y2HGold strain. No growth was obtained on quadruple-dropout medium, indicating that the two proteins do not interact detectably in yeast (Supplementary Fig. 3).

### Natural variation in *Tsc2* illuminates the genetic basis of tan spot susceptibility

We evaluated 390 *Triticum* accessions for reaction to ToxB and genotyped them for the presence/absence of a *Tsc2* allele (Supplementary Data 10–13). The accessions consisted of 112 hexaploid spring wheat and 58 winter wheat lines (*T. aestivum* ssp. *aestivum*), 99 tetraploid durum wheat lines (*T. turgidum* ssp. *durum*), and 121 tetraploid wheat relatives, including wild emmer [*T. turgidum* L. ssp. *dicoccoides* (Körn. ex Asch. & Graebn.) Thell.], cultivated emmer [*Triticum turgidum* L. ssp. *dicoccum* (Schrank) Thell.], and Persian wheat [*Triticum turgidum* L. ssp. *carthlicum* (Nevski) Á. Löve & D. Löve]. ToxB sensitivity was common among spring wheat (41/112, 37%) and durum (52/99, 52%) lines, but it was rare among the winter wheat lines (3/58, 5%) and the tetraploid relatives (2/121, 2%). Gene-specific PCR revealed that the entire *Tsc2* coding region was absent in 221 lines (57% of all lines), all of which were ToxB-insensitive.

**Fig. 7:**
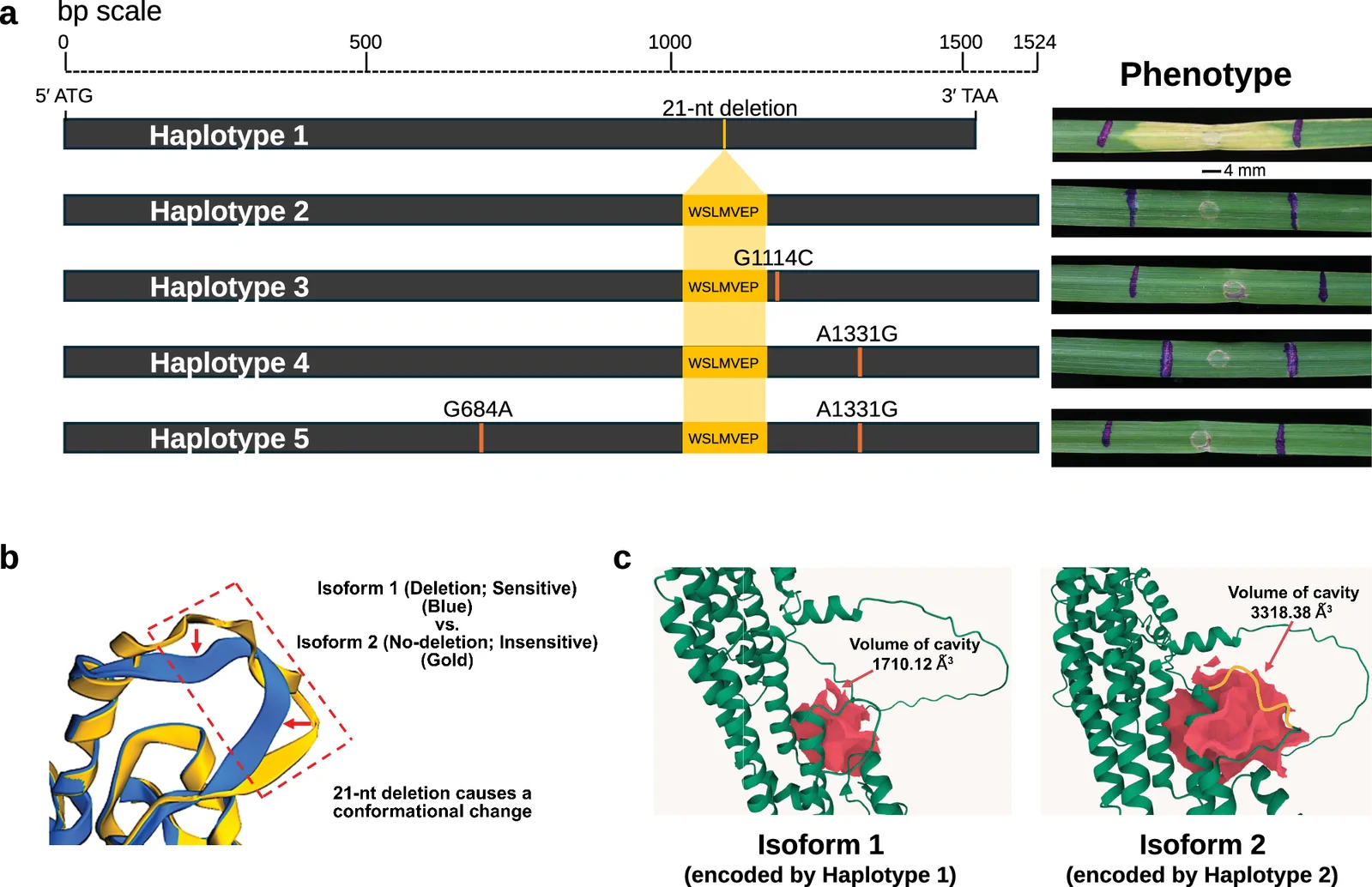
Structures and phenotypes of the five DNA haplotypes and corresponding protein isoforms of *Tsc2* identified among 177 wheat accessions. **a** Haplotype structures and the reactions they confer to infiltrations with ToxB. Haplotype 1 encodes the canonical ToxB-sensitive isoform and differs from Haplotypes 2–5 by a 21-nucleotide (nt) deletion. Haplotypes 2–5 share a 21-nt insertion encoding seven additional amino acids (WSLMVEP; highlighted in yellow). Haplotypes 3–5 harbor additional single-nucleotide substitutions (G1114C, A1331G, and G684A), indicated by orange bars and labeled above the haplotype schematic. Representative ToxB reactions of leaves for each isoform are shown to the right. **b** The portion of superimposed predicted protein structures of Isoforms 1 and 2, encoded by Haplotypes 1 and 2, respectively, showing the structural consequence of the 21-nt indel. **c** Protein cavity comparison in Isoform 1 vs Isoform 2. The central cavity is highlighted in red. The 21-nt insertion in Isoform 2, corresponding to the additional seven-amino acids (residues 370–376), is shown in yellow.

Among the 177 lines that retained an allele of *Tsc2*, Sanger sequencing resolved five haplotypes (Table 1, Fig. 7). The canonical ToxB-sensitivity allele (Haplotype 1) had a 21-nt deletion compared to all other haplotypes, which all conferred ToxB insensitivity. These 21 nts encoded amino acids WSLMVEP at positions 370–376. The deletion was present in 98 lines, all of which were sensitive to ToxB (Table 1, Supplementary Data 14). Haplotypes 1 and 2 differed only by the 21-nt indel, and the latter was specific to wild emmer wheat. Haplotypes 3, 4, and 5 retained the ancestral 21-nt segment and carried one or more single-nucleotide substitutions, and they were specific to durum wheat, cultivated emmer wheat, and common hexaploid wheat, respectively. Therefore, ToxB-mediated tan spot susceptibility in wheat appears to be conferred by the 21-nt deletion in the CAT-C domain of *Tsc2*, whereas complete loss of the gene or retention of the 21-nt segment results in resistance.

**Table 1:** *Tsc2* haplotype distribution across 177 *Triticum* accessions representing five subspecies

*Tsc2* haplotype	Length (bp)	ToxB reaction^b^	N^c^	Wheat class^a^				
				Common wheat	Durum wheat	Cultivated emmer	Wild emmer	Persian wheat
Haplotype 1	1503	S	98	44	52	0	0	2
Haplotype 2	1524	I	2	0	0	0	2	0
Haplotype 3	1524	I	11	0	11	0	0	0
Haplotype 4	1524	I	22	0	0	22	0	0
Haplotype 5	1524	I	44	44	0	0	0	0

^a^Common wheat: *Triticum aestivum*; Durum wheat: *Triticum turgidum* ssp. *durum*; Cultivated emmer: *Triticum turgidum* ssp. *dicoccum*; Wild emmer: *Triticum turgidum* ssp. *dicoccoides*; Persian wheat: *Triticum turgidum* ssp. *carthlicum*. Common wheat is hexaploid and includes both spring and winter types, and all others are tetraploid.

^b^Reaction to ToxB where S = sensitive; I = insensitive.

^c^*N* = Total number of accessions identified with each haplotype.

To examine the structural consequence of the indel, we compared the predicted protein structures of protein isoforms encoded by Haplotypes 1 and 2, which differ only for the seven-amino acid loop (370–376) encoded by the 21-nt indel. Presence of the seven residues nearly doubles the principal surface cavity (surface area 1755 Å^2^; volume 3318 Å^3^) (847 Å^2^; 1710 Å^3^) and raises the CavityPlus DrugScore (6626 versus 1714), without changing predicted binding energy (mean pKd≈6.95 in both) (Fig. 7; Supplementary Data 15).

### Emergence and spread of the *Tsc2* susceptibility allele

**Fig. 8:**
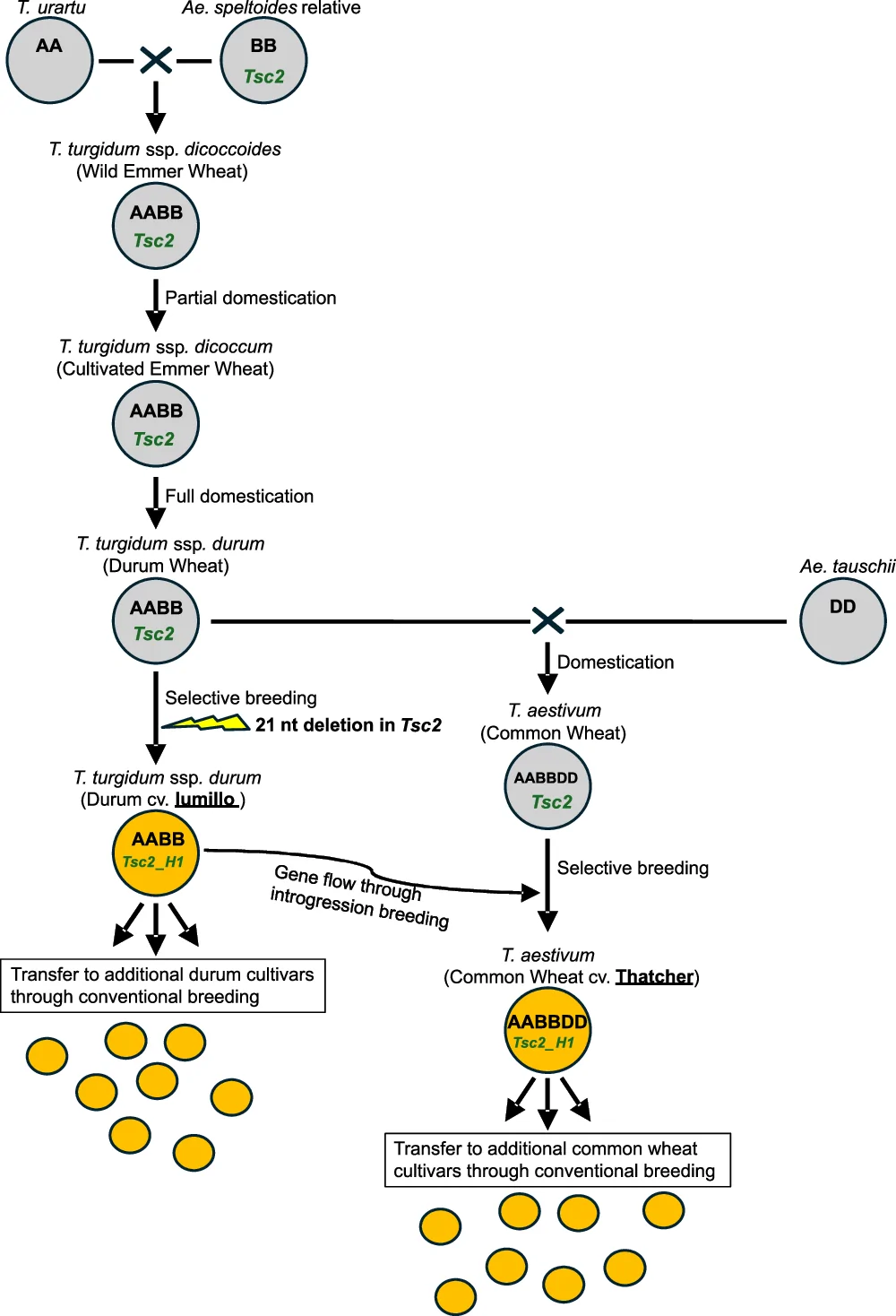
Evolution of wheat and the proposed formation and spread of the *Tsc2* allele conferring sensitivity to ToxB (Haplotype 1). The figure denotes the accepted evolutionary scenario giving rise to domesticated durum and common wheat. Gray circles indicate ToxB-insensitive (sub)species, and yellow circles indicate ToxB-sensitive species harboring the allele with the 21-nt deletion shown as *Tsc2_H1* (haplotype that results in amino acid Isoform 1). The point where the deletion occurred is indicated by the lightning bolt. Gray circles with ‘*Tsc2*’ in green are insensitive to ToxB but harbor alleles that give rise to Haplotypes 2–5, which all lack the 21-nt deletion.

Evaluation of the available genome sequences of the A- and D-genome diploid progenitors, AB tetraploids, and ABD hexaploids indicated that *Tsc2* homoeoalleles do not exist on the homoeologous chromosomes 2A or 2D. However, all B-genome relatives (*Ae. speltoides*, *Ae.sharonensis*) and progenitors (*T. turgidum* ssp. *dicoccoides*) with available genome sequences harbored a *Tsc2* allele that retained the 21‑nt segment deleted in the ToxB-sensitive allele (Haplotype 1). This indicates that the *Tsc2* allele that confers insensitivity to ToxB is the primitive form, and sensitivity was acquired upon deletion of the 21-nt segment (Haplotype 1). Because the allele that confers sensitivity to ToxB did not occur in any of the durum wheat progenitors and was more prevalent among durum cultivars than hexaploid wheat cultivars, we hypothesized that the deletion giving rise to the ToxB-sensitive allele occurred in a durum wheat cultivar and was inadvertently introduced into hexaploid wheat through conventional cross hybridization (Fig. 8).

To explore this hypothesis, we evaluated the pedigree of the oldest ToxB-sensitive hexaploid cultivar in our panel, which was Thatcher (Supplementary Data 10), released in 1935. Evaluation of the lines in the pedigree of Thatcher (Marquis/Iumillo//Marquis/Kanred) indicated that all were ToxB-insensitive except for Iumillo, which is a durum wheat cultivar released circa 1904 (Supplementary Data 10, 12, 14). This finding suggests that the 21-nt deletion, which resulted in the ToxB-sensitive allele, likely occurred first in a domesticated durum cultivar and was inadvertently transferred to common wheat cultivars such as Thatcher, where it was subsequently transferred to additional common wheat cultivars through conventional breeding practices.

### Tools for the development of tan spot-resistant wheat

Elimination of the *Tsc2* ToxB-sensitivity allele through conventional selective breeding or disruption of function through gene editing would lead to ToxB insensitivity and increased tan spot resistance. Here, we pursued both options to provide tools for wheat researchers to develop tan spot-resistant wheat.

**Fig. 9:**
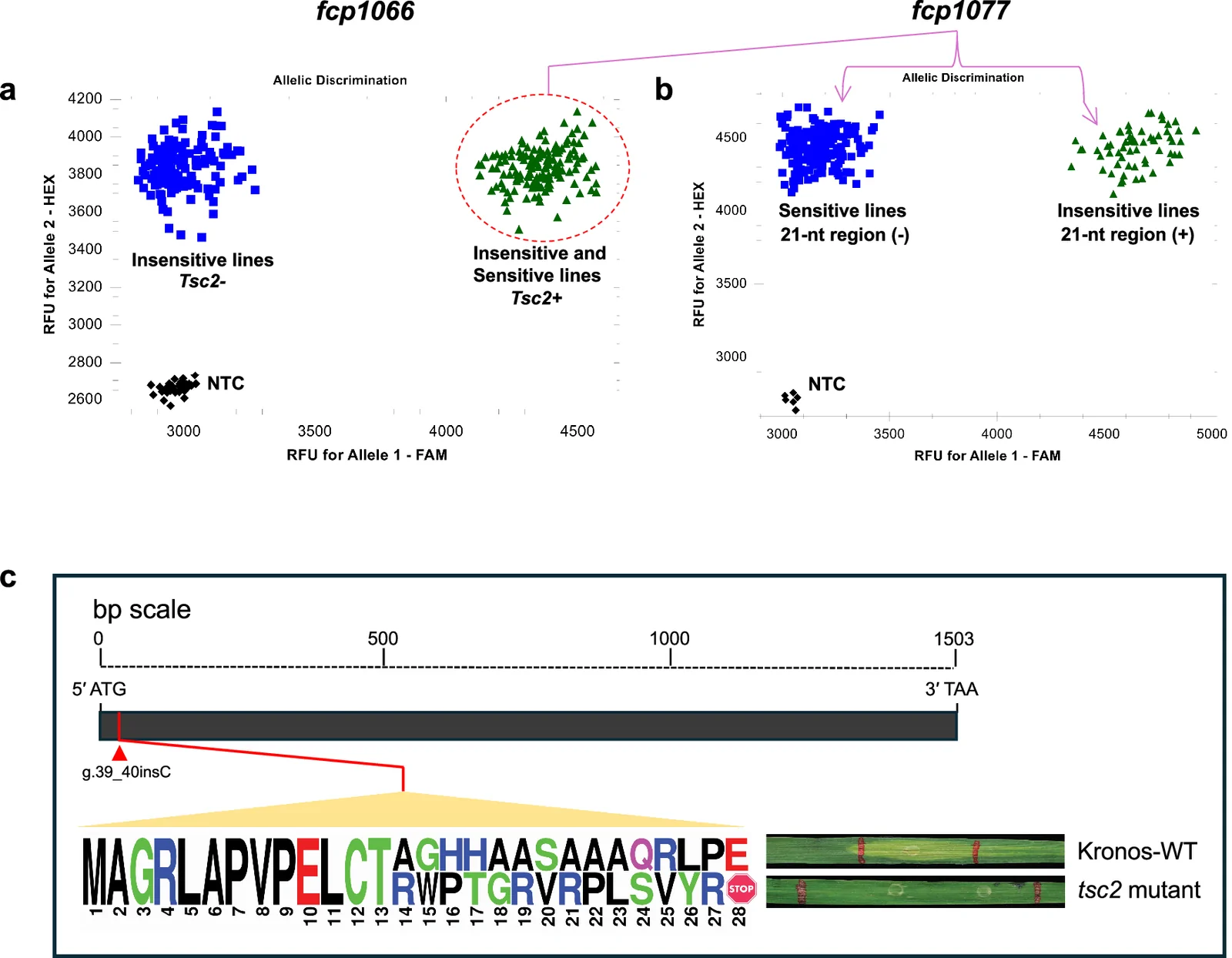
Marker-assisted and gene-editing tools developed for *Tsc2* elimination to confer tan spot resistance in wheat. **a**, **b** The two-step marker assay process for identification of ToxB-sensitive alleles in wheat. **a** Step #1 – Screening for the presence/absence of a *Tsc2* allele. Scatter plot of Global Durum Panel (GDP) lines genotyped with the fluorescence-based absent allele-specific amplification (FAASA) assay *fcp1066*. The fluorescence clusters separate lines with an absent allele of *Tsc2* (blue; *Tsc2*-) from lines having a *Tsc2* allele (green; *Tsc2*+). Non-template controls (NTC) are represented by black diamonds. **b** Step #2 – Screening of the green cluster from Step 1, which consists of lines all harboring a *Tsc2* allele, but a mixture of sensitive and insensitive haplotypes. Scatter plot of Global Durum Panel (GDP) lines genotyped with the FAASA assay *fcp1077*, which discriminates the 21-nucleotide (nt) indel in *Tsc2*. Clusters distinguish the deletion allele (−21 nt; sensitive; blue) from the ancestral allele (+21 nt; insensitive; green). **c** Gene-editing route to eliminate sensitivity. CRISPR/Cas9-mediated editing of *Tsc2* shows insertion of a cytosine after base position 39 (g.39_40insC), which induces a frameshift and introduces a premature stop codon. The sequence representation shows the predicted amino acids before and after the insertion: single letters correspond to the shared wild-type sequence upstream of the edit site, while stacked letters represent the divergence beyond the insertion point, with the upper row indicating the wild-type sequence and the lower row the CRISPR-induced mutant sequence that terminates prematurely. Leaf phenotype images on the right show ToxB sensitivity in wild-type Kronos (Kronos-WT) versus ToxB insensitivity in the *tsc2* mutant, confirming the functional disruption of *Tsc2*. The wild-type and CRISPR-derived amino acid sequences in (**c**) were visualized using the WebLogo 3 web application (https://weblogo.threeplusone.com). The stop icon: Created in BioRender. Seneviratne, S. (2026) https://BioRender.com/pkj2xsu.

To enable efficient elimination of *Tsc2* in breeding pipelines, we developed a two-step genotyping scheme based on the fluorescence-based absent allele-specific amplification (FAASA) strategy^42^. First, we designed a presence/absence marker (*fcp1066*) targeting the absent allele of *Tsc2* (Fig. 9a). Marker *fcp1066* was applied to three major wheat panels totaling 1483 lines: the Global Durum Panel (GDP; *n* = 470), a winter wheat (WW) panel (*n* = 235), and a spring wheat (SW) panel (*n* = 778)^43–45^. Consistent with prior reports, 66.2% of GDP lines^30^ and all WW lines were insensitive to ToxB^44^, whereas phenotyping of the SW panel performed in this study revealed that 96.0% were insensitive and 4.0% were sensitive (Supplementary Data 16–18). Across all three panels, *fcp1066* resolved samples into two separate non-overlapping fluorescence clusters, corresponding to lines lacking *Tsc2* (*Tsc2*-) and lines carrying a *Tsc2* allele (*Tsc2*+) of unknown haplotype (Fig. 9a). All lines classified as *Tsc2*- were insensitive to ToxB, yielding 100% concordance between the absent allele of *Tsc2* and ToxB insensitivity among these sets of diverse wheat germplasm.

Because *Tsc2*+ lines can carry insensitive haplotypes (lacking the 21-nt deletion) or the sensitive haplotype (with 21-nt deletion), we developed a second marker assay (*fcp1077*) to discriminate alleles at the 21-nt indel within the CAT-C domain. This assay robustly separated the deletion allele (−21-nt; ToxB-sensitive Haplotype 1) from the ancestral allele (+21-nt; ToxB-insensitive Haplotypes 2–5) among *Tsc2*+ lines (Fig. 9b). In the durum panel, for example, lines with the −21-nt allele corresponded to Haplotype 1 and were uniformly sensitive, whereas lines with the +21-nt allele carried Haplotype 3 and were insensitive (Table 1, Supplementary Data 14, 16).

In parallel, we used CRISPR/Cas9 as a proof-of-principle approach to disrupt *Tsc2* function in Kronos. Using two guide RNAs targeting adjacent sites near the 5′ end of the *Tsc2* coding sequence, we recovered a single-base insertion allele (g.39_40insC), which resulted in a frameshift and premature stop codon (Fig. 9c). This edited allele was recovered from one transgene-positive T_0_ plant obtained after *Agrobacterium*-mediated transformation of 100 Kronos immature embryos (Supplementary Data 19). The predicted truncated protein lacked almost the entire Exo70 core. Following self-pollination, homozygous T_1_ plants carrying the edited allele showed complete loss of ToxB-induced chlorosis compared to wild-type Kronos, confirming that CRISPR/Cas9-mediated disruption of *Tsc2* is sufficient to convert a sensitive line into an insensitive one. Together, these marker-assisted and gene-editing tools provide complementary, breeder-ready strategies to eliminate *Tsc2*-mediated tan spot susceptibility in wheat.

## Discussion

The identification of *Tsc2* as a member of the Exo70 subunit family marks a significant expansion of the known classes of disease susceptibility genes in plants. Previously characterized NE-recognizing susceptibility genes in wheat include *Snn1*^46,47^ and *Snn3*^48,49^ in the wheat-*Parastagonospora nodorum* system, *Tsc1*^22^ in the wheat-*P. tritici-repentis* system, and *Tsn1*^17^, which operates in both systems. Snn1 (a wall-associated kinase; WAK) and Tsc1 (LRR-RLK) are both membrane-spanning proteins, and the extracellular domain of the former has been shown to interact directly with the corresponding NE, suggesting that these two interactions function in PTI pathways. In contrast, Snn3 (PK-MSP; major sperm protein) and Tsn1 (PK-NLR) are intracellular proteins that do not interact directly with their corresponding NEs but likely recognize them through intermediates, indicating they likely elicit PCD through ETI. Common among the above-mentioned genes is that they all encode PK domains, presumably involved in signaling, and they contain other integrated domains likely involved in protein-protein interactions, such as LRR and MSP domains^21,49^. In contrast, *Tsc2* encodes an Exo70 subunit of the exocyst complex, a fundamentally different protein class lacking any obvious receptor or enzyme activity. This finding broadens the paradigm of NE-triggered susceptibility beyond the typical perception-type genes (e.g., WAK, NLR, PK-MSP, LRR-RLK) and underscores the ability of necrotrophic pathogens to hijack diverse host proteins to induce susceptibility.

*Tsc2* resides in the grass-specific Exo70FX clade, a lineage that emerged after the monocot-dicot split and proliferated in Poales^40,50^. The biological specialization of many Exo70FX members remains unclear, but recent studies suggest roles in plant immunity^40,51^. In barley, an Exo70FX protein (HvExo70FX12) was found to be indispensable for resistance against stripe rust (*Puccinia striiformis*), acting in concert with an LRR-RLK to mediate race-specific immunity^40^. This finding implies that certain Exo70FX isoforms have evolved to partner with immune receptors or pathways, perhaps as adaptively evolved “secretory hubs” in defense. Exo70FX members have also been identified as integrated domains in NLR proteins, suggesting a role in pathogen recognition leading to ETI^52^. The function of the Exo70FX15 protein encoded by *Tsc2* is expected to function essentially the same as Exo70FX12 in barley, i.e., it functions to induce PCD and a ‘defense response.’ However, because Ptr is a necrotroph, it is equipped to thrive in such an environment and thereby exploits the host response to induce susceptibility. Interestingly, phylogenetic analyses show that the wheat Exo70FX15 and barley Exo70FX12 proteins are closely related sister variants that recently diverged from Exo70FX11 within the FX clade. Their repeated duplication and subsequent neofunctionalization in Triticeae likely stem from selection pressures imposed by pathogens^40,51^.

Classical Exo70 proteins typically function at or near the plasma membrane, where phospholipid-binding domains contribute to vesicle tethering and exocyst assembly^39,41,53^. In many plants, Exo70 proteins (Exo70A1 and Exo70B2) cluster at the cell periphery and mark sites of exocyst activity^41,54^. By contrast, Tsc2 lacks the CorEx and CAT-A domains, which are required to drive the membrane-bound vesicles to the plasma membrane^39^. A parallel observation was made previously^40^ for barley HvExo70FX12, which lacks the CorEx and CAT-A domains integral to exocyst assembly, indicating a specialized receptor-kinase immunity pathway rather than typical exocytosis^51^. We found that an N-terminal fusion of GFP with Tsc2 localizes to the nucleus and cytoplasm and does not appear to localize to the plasma membrane, indicating that it may operate in intracellular signaling processes rather than direct pathogen contact. Because ToxB remains in the apoplast^34^, it is possible that an unknown wheat RLK or receptor-like protein perceives ToxB at the plasma membrane and subsequently recruits Tsc2 through direct or indirect interaction, leading to downstream defense signaling via regulation of nuclear transcription and ultimately PCD.

When hexaploid wheat (*T. aestivum*) formed ~10,000 years ago from the hybridization of an AB tetraploid wheat with *Ae. tauschii*, it likely acquired the primitive *Tsc2* allele, which contained the critical 21-nt segment and was insensitive to ToxB. Because the ToxB-sensitive allele (Haplotype 1) is much more prevalent in durum cultivars (~32%) than in the durum progenitors (0%) and hexaploid wheat cultivars (<5%), we hypothesize that the critical 21-nt deletion that gave rise to the ToxB-sensitive allele occurred in a domesticated durum cultivar and was subsequently introgressed into modern hexaploid wheat varieties through conventional breeding practices. Our finding that the hexaploid spring wheat cultivar Thatcher likely obtained the *Tsc2* susceptibility allele from the ToxB-sensitive durum variety Iumillo supports this hypothesis. Iumillo was used in the development of Thatcher to transfer stem rust resistance^55^, and ToxB sensitivity conferred by Haplotype 1 of the *Tsc2* allele was concomitantly transferred. Thatcher became a dominant cultivar in the Great Plains of the USA and Canada for several decades because of its good levels of stem rust resistance, and it was widely used in HRSW breeding programs^56^. Therefore, it is possible that Thatcher served as a hexaploid source for the *Tsc2* susceptibility allele to be incorporated into other HRSW lines. However, this is only one scenario, and it is possible that additional events occurred that moved the susceptible allele from durum to hexaploid wheat lines.

For decades, vulnerability to tan spot remained hidden; partially because of intensive tillage and burning of crop residues that kept residue‑borne pathogens in check^12,13^, and Ptr races carrying ToxB were not prevalent. The shift to reduced tillage and continuous cropping in the 1970s changed that calculus, boosting inoculum and exposing the functionality of *Tsc2* in many Thatcher derivatives^5,25^. Today, ToxB sensitivity reaches 4–9% in spring wheat panels^44,57^, mirroring the global footprint of Thatcher germplasm, and in some regions of the world, ToxB sensitivity has been shown to be present in more than 30% of durum lines and 20% of winter wheat lines^58^. Thus, a seven‑codon deletion that likely arose in durum and rode into hexaploid wheat on the coattails of rust resistance now poses a major foliar disease threat.

Most modern wheat breeders employ genomic and marker-assisted selection using high-throughput platforms consisting of thousands of markers. Together, marker assays *fcp1066* and *fcp1077* provide a simple two-step process for breeders to characterize their lines for *Tsc2*. First, they can use the *fcp1066* assay to identify all *Tsc2*- lines as inherently ToxB-insensitive. If deeper selection is desired, they can then screen the *Tsc2*+ lines with the *fcp1077* assay to identify lines carrying the deleterious −21-nt susceptibility allele. The ability to identify lines lacking *Tsc2* with 100% accuracy eliminates the need for time-consuming infiltration assays, ensuring that only lines insensitive to ToxB advance in breeding pipelines. These marker assays can be readily incorporated into established marker-assisted and genomic selection pipelines and therefore provide a promising solution for most wheat breeding programs to minimize tan spot susceptibility. In this capacity, our study provides a cost-effective and straightforward strategy to substantially improve tan spot resistance.

We also demonstrated that gene editing can be used to effectively ‘turn off’ *Tsc2* to generate a ToxB-insensitive allele. Should gene-editing methods in wheat become socially and politically accepted, this technology may prove efficient for disrupting disease susceptibility genes in wheat cultivars in a targeted and focused manner, as opposed to conventional breeding practices that run the risk of introducing undesirable alleles at other loci, just as the ToxB-sensitive allele of *Tsc2* was inadvertently introduced into Thatcher wheat. Because *Tsc2-*absent alleles are quite common in nature, it is unlikely that the present *Tsc2* alleles of any haplotype have a critical function. However, if it were to be discovered in the future that a particular *Tsc2* allele was beneficial under certain conditions, gene editing may serve as an important tool for the generation of novel alleles that may serve the newly discovered beneficial function as well as insensitivity to ToxB. Allele engineering could also take into account the ability to develop a high-throughput marker for that allele.

Overall, this work shows that a neofunctionalized plant exocyst component underwent a mutation in the form of a 21-nt deletion that allowed it to be hijacked by a pathogen effector, resulting in the exacerbation of disease. Not only does this work shed light on the role of *Tsc2* in ToxB-induced tan spot susceptibility, but it also broadens our understanding of host targets exploited by necrotrophic pathogens. By illustrating how a single exocyst subunit can function as a susceptibility factor, this study also highlights the remarkable evolutionary plasticity of the exocyst complex and suggests that further investigation of other Exo70FX subfamily members will likely deepen our understanding of plant disease resistance and susceptibility mechanisms. From a practical perspective, the knowledge and tools developed in this work will allow wheat breeders to efficiently develop tan spot-resistant cultivars in the present and future.

## Methods

### Plant materials

A durum wheat population of 149 RILs (KG RIL population) derived from Kronos (ToxB-sensitive) × Gredho (insensitive) was used for initial and saturation mapping of *Tsc2*. The same cross was made to develop an F_2_ population for high-resolution mapping. A total of 7002 F_2_ plants were evaluated for reaction to ToxB, and 1492 insensitive plants were selected for genotyping. Kronos TILLING lines^38^ and the hexaploid wheat cultivar Prosper were used for the evaluation of mutations as described below. For genetic complementation, we used the hexaploid wheat cultivar Fielder as the transformation recipient, and the durum cultivar Kronos was used for gene editing and transcription experiments.

The hexaploid wheat cultivar Jimai 22 was used for subcellular localization experiments. For natural allelic variation analyses and marker validation, we evaluated three germplasm panels totaling 1483 cultivated lines: a global durum panel (GDP, *n* = 470), a spring wheat panel (HRSW, *n* = 778), and a winter wheat panel (WW, *n* = 235) (Supplementary Data 16–18). We also examined a diverse set of 121 wild and domesticated tetraploid relatives that included *T. turgidum* spp. *dicoccoides* (*n* = 6), *dicoccum* (*n* = 109), and *carthlicum* (*n* = 6) (Supplementary Data 13).

### Plant growth and phenotyping

All experiments were conducted in a greenhouse or growth chambers at 21 °C with a 12 h photoperiod. Seeds of all lines were sown in Super Cell cone containers (Stuewe & Sons) filled with Sunshine SB100 mix (Sun Gro Horticulture) and supplemented with Osmocote Plus (15-9-12; Scotts). ToxB cultures were produced, and plants were infiltrated and scored as described previously^30^. Infiltration reactions are evaluated at 3–5 days post-infiltration on a scale of 0–3 where 0 shows no reaction, 1 shows a slight but visible chlorosis, 2 shows a visible chlorosis without tissue collapse, and 3 shows complete tissue collapse.

### Genomic DNA extraction, genotyping, and linkage mapping in the KG RIL population

DNA was extracted as described previously^59^, and genotyping was performed at the USDA-ARS Small Grains Genotyping Lab (Fargo, ND) using the Illumina Infinium iSelect 90 K SNP array^60^. GenomeStudio v2.0 was used for clustering and genotype calling. Linkage analysis was carried out in MapDisto v2.1.7 as described previously^61^.

### Saturation mapping and high-resolution mapping

Genomic DNA for all assays was extracted from seedling leaves as described above. SSR markers were mined from the genomic sequence flanking the *Tsc2* region in the *T. turgidum* cv. Kronos and the synthetic hexaploid W7984 assemblies^37,62^. SSRs were identified with Krait v1.3.3, and primers were designed in Krait/Primer3 (https://www.ncbi.nlm.nih.gov/tools/primer-blast/)^63^. Polymorphic SSRs were genotyped across the KG RILs and added to the linkage map. KASP assays were developed from SNPs differentiating Kronos and Gredho using publicly available exome-capture data (WheatCAP project 2017_WheatCAP_UCD; https://wheat.triticeaetoolbox.org/) aligned to the Chinese Spring reference (IWGSC RefSeq v1.0; https://graingenes.org/). Assays were designed with PolyMarker^64^ to ensure genome specificity. Primer sequences and all SSR/KASP marker positions are listed in Supplementary Data 3.

Kronos × Gredho F_2_ plants were infiltrated with ToxB and classified as sensitive or insensitive. A total of 1492 plants were insensitive and therefore homozygous recessive at *Tsc2*. Flanking markers *fcp1028* and *fcp1033* were used to screen the 1492 F_2_ plants to identify crossovers, and all recombinants were genotyped with the full interval marker set.

### Comparative genomics

We analyzed the candidate gene region in 12 sequenced lines: four ToxB-sensitive lines (Kronos, Simeto, Glenn, SY Mattis v1.0) and eight ToxB-insensitive lines (ArinaLrFor v3.0, CDC Landmark v1.0, Chinese Spring v2.1, Fielder, Jagger v1.0, Julius v1.0, LongReach Lancer v1.0, Svevo v1.0) (Supplementary Data 5). Public assemblies were obtained from GrainGenes (https://graingenes.org/), Wheatomics (http://wheatomics.sdau.edu.cn/), and Ensembl Plants (https://plants.ensembl.org/), and scaffold assemblies for Simeto and Glenn were provided by USDA-ARS (Fargo, ND). Where available, existing GFF3 annotations were used. For unannotated assemblies, gene models were predicted with AUGUSTUS (https://bioinf.uni-greifswald.de/augustus/) using wheat parameters. Predicted coding sequences were translated and functionally annotated using the NCBI Conserved Domain Database and InterProScan v5.52-86.0. Following established criteria^23^, features <500 bp or lacking Pfam support at *E* < 1 × 10^−5^ were classified as pseudogenes/fragments and excluded from downstream comparison.

Orthology and presence-absence were assessed by BLAST (nucleotide and protein) against Kronos (genome v1.1) gene models^37^, recording percent identity and query coverage. Multiple sequence alignments were used to examine large polymorphisms (insertions/deletions, truncations) within coding regions. Genes conserved across all 12 genomes were recorded as shared; genes present and intact in all sensitive lines but absent or structurally disrupted in insensitive lines were flagged as candidates for *Tsc2* (Supplementary Data 5).

### EMS and TILLING mutant screening

Kronos TILLING lines^38^ were queried for nonsense or predicted deleterious missense variants in *TrturKRN2B01G009130* (LRR-RLK) (*n* = 19 lines) and *TrturKRN2B01G009140* (*TtExo70FX15*) (*n* = 10 lines) (Supplementary Data 6 and 7). Mutant families were grown and assayed for ToxB reaction. Candidate lines were verified by Sanger sequencing of the target amplicon. The insensitive families were re-tested in the next generation to confirm the phenotype. A Prosper EMS population of 1050 M_2_ families, previously developed^22^, was evaluated for sensitivity to ToxB. Fourteen seeds from each family were infiltrated with ToxB, and *TaExo70FX15* was sequenced from insensitive plants using Sanger sequencing and aligned with wild-type Prosper sequences. Variants in Kronos TILLING and Prosper EMS lines were classified as synonymous, missense, or nonsense. Missense variants were evaluated with SIFT^65^ and inspected with Missense3D (https://missense3d.bc.ic.ac.uk/) and DynaMut2 (https://biosig.lab.uq.edu.au/dynamut2/) for predicted structural impacts.

### *Agrobacterium*-mediated transformation and functional complementation of *Tsc2*

A 6468-bp genomic fragment comprising the complete *Tsc2* open reading frame plus 2734 bp upstream and 2231 bp downstream sequences was PCR-amplified from Kronos genomic DNA with Phusion® High-Fidelity polymerase (New England Biolabs). Primers were designed with overhangs compatible with the In-Fusion® HD Cloning system (Takara Bio USA, Inc.). The binary vector pCAMBIA1300-ubi-GRF-GIF^66^ was linearized with *Kpn*I and *Spe*I (New England Biolabs) and purified using the Wizard® SV Gel and PCR Clean-Up System. The purified insert was then ligated into the linearized vector using the In-Fusion® Snap Assembly (Takara Bio USA, Inc.) according to the manufacturer’s protocols. The recombinant plasmid was introduced into NEB 5-alpha Competent *E. coli* cells (New England Biolabs) via heat shock transformation, and colony PCR was used to verify successful transformants. Plasmid DNA from confirmed clones was extracted using the QIAprep Spin Miniprep Kit (Qiagen). The complete construct was then validated by whole-plasmid sequencing (Eurofins Genomics) to ensure the correct insertion of the 6468 bp fragment. The validated recombinant plasmid was subsequently introduced into *Agrobacterium tumefaciens* strain AGL1T^67^ by electroporation.

Single colonies were verified by PCR, propagated in LB (kanamycin + rifampicin), and stored in 15% glycerol at −80 °C. Transformation was performed using a previously described protocol^68^, with minor adjustments. Briefly, immature Fielder embryos (~14 d post-anthesis) were excised after surface sterilization and inoculated with AGL1T containing the construct (OD_600_ ~ 0.5, 100 µM acetosyringone, 0.05% Silwet L-77). After 3 days of co-cultivation at 24 °C in the dark, embryos were rested for 5 days on wheat callus-induction medium (WCI). Hygromycin selection was then carried out on WCI containing 15 mg/L hygromycin for 2 weeks in the dark, followed by selection on WCI supplemented with 30 mg/L hygromycin for an additional 3 weeks, consisting of 2 weeks in the dark and 1 week under weak light conditions. Hygromycin-resistant calli with green foci were transferred to wheat regeneration medium (WRM + 0.5 mg/L Zeatin); recalcitrant calli were transferred to WRM supplemented with 1 mg/L zeatin. Shoots with roots were hardened on hygromycin (15 mg/L) rooting medium, then transplanted to vermiculite and finally to soil (22 °C, 16 h light). T_0_ plants were screened at the two- to three-leaf stage by leaf infiltration with ToxB infiltration of individual leaves from each tiller (described above). Following phenotypic scoring, leaf tissue from each infiltrated tiller was collected, and the presence of the transgene was verified by PCR with *Tsc2*-specific primers (Supplementary Data 19) and Sanger sequencing.

### RNA isolation, first-strand cDNA synthesis, and RACE cloning

To investigate the expression pattern of *Tsc2* across wheat tissues, samples were collected from the durum wheat cultivar Kronos. Leaf and root tissues were harvested from two-week-old seedlings, stem tissue was collected at Feekes growth stage 7 (two nodes detectable; https://nutrien-ekonomics.com/infographics/wheat-growth/), and immature spike tissue was collected at Feekes growth stage 9 (flag leaf ligule just visible).

Total RNA was extracted and cDNA prepared from Kronos tissues as described in Zhang et al.^49^. RT-PCR amplification was performed using gene-specific primers for *Tsc2* and the housekeeping gene *GAPDH*, which served as an endogenous control (primer sequences listed in Supplementary Data 20). The *GAPDH* primers were designed to distinguish cDNA from genomic DNA, yielding a 187-bp product from cDNA and a 357-bp product from genomic DNA^17^. PCR products were resolved by agarose gel electrophoresis to verify amplification specificity. Detection of the expected cDNA-sized *GAPDH* amplicon confirmed successful reverse transcription.

For RACE cloning, *Tsc2* coding sequences were amplified with gene-specific primers (Supplementary Data 20), purified from 1.5% agarose gels, and recovered with the Wizard® SV Gel & PCR Clean-Up kit (Promega). 5′- and 3′-UTRs were evaluated using the SMART RACE (SMARTer RACE 5′/3′ Kit; Takara Bio USA). Reads were assembled in Geneious Prime 2025.1.3 (https://www.geneious.com) to obtain the full-length *Tsc2* cDNA, delineate the 5′/3′ UTRs, and define exon-intron boundaries by alignment to the genomic sequence.

### qRT-PCR assay

cDNA from Kronos and DWRC-1014 leaves was serially diluted (1:2, 1:4, 1:8, 1:16, 1:32). Reactions (10 μL) contained 5 μL SYBR^TM^ Select Master Mix (Thermo Fisher Scientific), 0.5 μM each primer (for *Tsc2* or *GAPDH*), and 2 μL diluted cDNA, and were run on a Bio-Rad CFX384^TM^ system with: 95 °C 2 min; 40 cycles of 95 °C 15 s, 60 °C 1 min; followed by a melt curve (65-95 °C, 0.5 °C increments). Standard-curve slopes yielded amplification efficiencies within 90–110% for both targets, permitting ΔΔCq-based relative quantification in the time course.

For the temporal experiment, secondary leaves of two-week-old Kronos and DWRC-1014 plants were infiltrated with ToxB or YPD media (mock). Tissue was collected at 6, 24, 48, and 72 hpi. For each time point and treatment, 5–7 biological replicates were analyzed, with ≥3 technical replicates per sample. Cq values were averaged across technical replicates and normalized to *GAPDH* (ΔCq). Cq values were exported from Bio-Rad CFX Maestro 2.3 (Version: 5.3.022.1030) and analyzed by the ΔΔCq method. *Tsc2* was normalized to *GAPDH*, which showed stable expression across samples. The 6 hpi YPD group was used as the calibrator for both Kronos and DWRC-1014, and fold change was calculated as 2^-ΔΔCq^. Group means and SEM were visualized from biological replicates (5–7 per treatment and time point). Between-treatment differences at each time point were evaluated with two-sided Welch’s *t*-tests in ‘*scipy.stats.ttest_ind*’ (SciPy v1.17.0; Python v3.12.13), with unequal variances assumed (*equal_var=False*).

### 3D structure of Tsc2 and phylogenetic analysis

The Tsc2 protein was modeled with AlphaFold2 via the ColabFold v1.5.2-patch pipeline^69,70^ using default settings, and the highest-confidence model (pLDDT and PAE) was used for analysis. Protein structures were generated to predict the wild-type TtExo70FX15 (Tsc2) protein, and missense mutations were mapped onto the predicted structure to visualize their spatial positions (Supplementary Figs. 1 and 2). To place Tsc2 within the Exo70 structural framework, the predicted model was aligned to yeast Exo70 (PDB: 2B1E) and *Arabidopsis* EXO70A1 (PDB: 4RL5) using TM-align (https://www.rcsb.org/alignment), and superpositions were examined in UCSF ChimeraX v1.6.1 (https://www.cgl.ucsf.edu/chimerax/). Exo70 subdomains (CorEx, CAT-A to CAT-D) were assigned by transferring domain boundaries from yeast Exo70 onto the aligned Tsc2 structure and refining these boundaries using MAFFT-DASH structure-guided alignments and ChimeraX Matchmaker.

To evaluate the structural consequences of natural variation, additional AlphaFold2 models were generated for the +21-nt (ancestral) and −21-nt (deletion) alleles. Missense mutations identified in TILLING and EMS populations were mapped onto the wild-type TtExo70FX15 structure to visualize their spatial positions. Missense effects were assessed with SIFT^65^, Missense3D (https://missense3d.bc.ic.ac.uk/), and DynaMut2 (https://biosig.lab.uq.edu.au/dynamut2/). The impact of the 21-nt indel on protein architecture was further examined using cavity predictions from CavityPlus with default parameters, and for each allele, the highest-scoring cavity was recorded (volume, surface area, DrugScore). All structural visualizations were prepared in UCSF ChimeraX v1.6.1.

For phylogenetic analysis, Tsc2 was incorporated into the Exo70FX11/FX12/FX15 data sets from Holden et al.^40^. Sequences were aligned with MAFFT-DASH v7.520 using structure-based alignment, filtered to remove redundant sequences, and trimmed to exclude poorly aligned positions. Maximum-likelihood trees were inferred using RAxML v8.2.12 with the GAMMA model of rate heterogeneity and 1000 bootstrap replicates. Phylogenies were visualized and annotated using iTOL v7.3 (https://itol.embl.de/).

### Subcellular localization of Tsc2 protein

Transient expression of an N-terminal GFP fusion of Tsc2 (35S::pAN580-Tsc2-GFP) was outsourced to Lifeasible (Shirley, NY, USA; www.lifeasible.com). The Tsc2 coding sequence was cloned into the pAN580 vector and verified by sequencing. For co-localization analysis, the Tsc2-GFP construct was co-transformed with a nuclear-marker plasmid encoding an NLS-mKATE fusion into mesophyll protoplasts isolated from 10- to 15-day-old seedlings of wheat cv. Jimai 22. The NLS peptide sequence was MDPKKKRKV. A GFP-only construct co-transformed with the same NLS-mKATE nuclear marker was used as a control. Protoplasts were transformed by PEG-mediated uptake, incubated for 18–24 h at 24 °C, and examined by laser-scanning confocal microscopy^49^. GFP, NLS-mKATE, chlorophyll autofluorescence, and bright-field channels were collected separately. Fluorescence intensity profiles were generated from the GFP and mKATE channels along transects crossing the nuclear-marker-positive region and adjacent cytoplasm. Profiles were evaluated for five independent Tsc2-GFP-transformed protoplasts and one GFP-only control.

### Yeast two-hybrid

Assessment of the physical interaction between Tsc2 (TtExo70FX15) and ToxB was subcontracted to Lifeasible (Shirley, NY, USA; www.lifeasible.com) and followed the Matchmaker Gold Yeast Two-Hybrid System protocol detailed by Zhang et al.^49^. In brief, ToxB cDNA was fused to the GAL4 DNA-binding domain in pGBKT7 (bait), while Tsc2 cDNA was fused to the GAL4 activation domain in pGADT7 (prey). Auto-activation and toxicity of the bait construct were evaluated prior to interaction testing. Bait and prey plasmids were co-transformed into the Y2HGold yeast strain, and transformants were selected on SD/-Leu/-Trp medium. Protein-protein interactions were assessed using the HIS3, ADE2, and lacZ reporter genes.

### Allelic variation and haplotype analysis

The *Tsc2* coding region was PCR-amplified from 390 *Triticum* accessions (Supplementary Data 10–13) using gene-specific primers spanning the full ORF (Supplementary Data 20) to determine the presence/absence of *Tsc2* alleles. Amplicons were sequenced from the 177 accessions that harbored a *Tsc2* allele. Reads were assembled in Geneious Prime to produce high-quality consensus sequences, translated, and aligned with MAFFT. Haplotypes were defined by the presence/absence of the 21-nt indel in CAT-C and by nonsynonymous SNPs (Supplementary Data 14).

### Development and evaluation of diagnostic FAASA marker assays

Two KASP-based FAASA multiplex assays were developed to enable high-throughput genotyping of *Tsc2*: *fcp1066*, which detects the presence/absence of the *Tsc2* gene, and *fcp1077*, which targets the diagnostic 21-nt indel within the gene. Both assays were designed using the FAASA framework^42^, in which a four-primer system is used to simultaneously amplify the target region and a conserved internal control. Each assay consisted of a pair of *Tsc2* target-specific primers (TSPs) and a pair of primers targeting the wheat *Q* gene (core gene-specific primers, CGSPs). The TSP forward primer carried a 5′ FAM tail, and the CGSP forward primer carried a 5′ HEX tail, enabling fluorescence-based discrimination of *Tsc2* and the internal control. Primer sequences for both markers are listed in Supplementary Data 20. Reactions were assembled and run according to the FAASA cycling conditions recommended by Running et al.^42^.

For *fcp1066*, samples producing only the HEX signal were scored as *Tsc2*-absent, whereas samples producing both HEX and FAM signals were scored as *Tsc2*-present. This marker was validated across all three germplasm panels described above (GDP, WW, SW; *n* = 1483). Marker *fcp1077* was designed to discriminate the 21-nt deletion (ToxB-sensitive) from the ancestral 21-nt insertion (ToxB-insensitive) within *Tsc2*, using the same FAASA multiplex design. Validation was performed using the durum panel, where allele calls matched the ToxB phenotypes with complete concordance. Because the indel is conserved across wheat subgenomes and the assay relies on the same core FAASA architecture as *fcp1066*, *fcp1077* is expected to perform robustly in additional germplasm.

### Gene editing using CRISPR/Cas9

CRISPR/Cas9 editing of *Tsc2* was carried out using the pCBC-MT1T2 gRNA assembly system^71^ and the JD633 CRISPR/Cas9 vector containing the GRF-GIF cassette^66^. Two gene-specific target sequences located near the 5′ end of *Tsc2* were incorporated into the MT1 and MT2 gRNA modules by PCR, and the resulting 966-bp product was gel-purified and assembled into JD633 using Golden Gate cloning^72^. All cloned constructs were verified by whole-plasmid nanopore sequencing (https://eurofinsgenomics.com) prior to *Agrobacterium* transformation. The validated CRISPR/Cas9 plasmid was introduced into *A. tumefaciens* strain AGL1T by electroporation, and single colonies were verified by PCR. Wheat transformation was performed in Kronos following the same *Agrobacterium*-mediated protocol described above for functional complementation, using identical embryo preparation, co-cultivation, selection, regeneration, and acclimation conditions. A total of 100 Kronos immature embryos were inoculated/co-cultivated with *Agrobacterium*, which yielded two regenerated T_0_ plants derived from independent calli. PCR screening of the CRISPR/Cas9 construct identified one transgene-positive T_0_ plant (Supplementary Data 20).

Regenerated T_0_ plants were genotyped by PCR using primers flanking the CRISPR target region, and amplicons were sequenced to identify editing events. Phenotyping of T_0_ leaves by infiltration with ToxB (as described above) was performed in parallel to assess sensitivity. An edited allele carrying a single-base C insertion at genomic position g.39_40, relative to the *Tsc2* coding sequence, was recovered in the transgene-positive T_0_ plant. This g.39_40 mutation produced a frameshift and premature stop codon in *Tsc2*. The edited T_0_ plant was self-pollinated, and five T_1_ progenies were screened using the same flanking primers and Sanger sequencing. T_1_ plants were phenotyped with ToxB, and sequencing confirmed that two homozygous edited plants were insensitive, whereas three plants carrying the wild-type *Tsc2* allele remained sensitive to ToxB.

### Reporting summary

Further information on research design is available in the Nature Portfolio Reporting Summary linked to this article.

## Supplementary information

Supplementary Information

Description of Additional Supplementary Information

Supplementary Data

Reporting Summary

Transparent Peer Review file

## Source data

Source Data

## Data Availability

All data supporting the findings of this study, including source data, are available within the article and its Supplementary Information files. The *Tsc2* haplotype sequences generated from 177 accessions have been deposited in GenBank (https://www.ncbi.nlm.nih.gov/genbank/) under accession numbers PV543977-PV544011, PV544013-PV544024, PV544026-PV544057, PV544059-PV544080, PV544082-PV544091, PV544093-PV544116, PV544118-PV544138, and PV544140-PV544160. All accessions can be accessed using https://www.ncbi.nlm.nih.gov/nuccore/XXXXXXXX and replacing the X’s with the specific accession numbers above. All genetic materials generated and used in this study are available from the corresponding author upon reasonable request and completion of a material transfer agreement (MTA). Source data are provided with this paper.
