# Supplementary material for: The hijacking of a wheat Exo70-like protein by a fungal pathogen triggers disease
Source: Nat Commun. 2026 Sep 10;17:10713. doi: 10.1038/s41467-026-77720-9 (PMC13658248; doi:10.1038/s41467-026-77720-9)

The hijacking of a wheat *Exo70*-like protein by a fungal pathogen triggers disease

Singh *et al.*

Supplementary Figure 1. Leaf infiltrations and TtExo70FX15 (Tsc2) protein structure visualizations for Kronos wild type (WT), four ToxB-insensitive Kronos TILLING mutants with missense mutations, and one Kronos TILLING mutant with a missense mutation that was ToxB-sensitive. Protein structures depict the AlphaFold-predicted wild-type TtExo70FX15 (Tsc2) protein, with missense mutation sites highlighted to indicate their spatial positions. The locations of the point mutations are shown in red and labeled next to orange arrows. Representative ToxB infiltration reactions for each mutant are shown on the right. Plants were each infiltrated with ToxB at least three times.

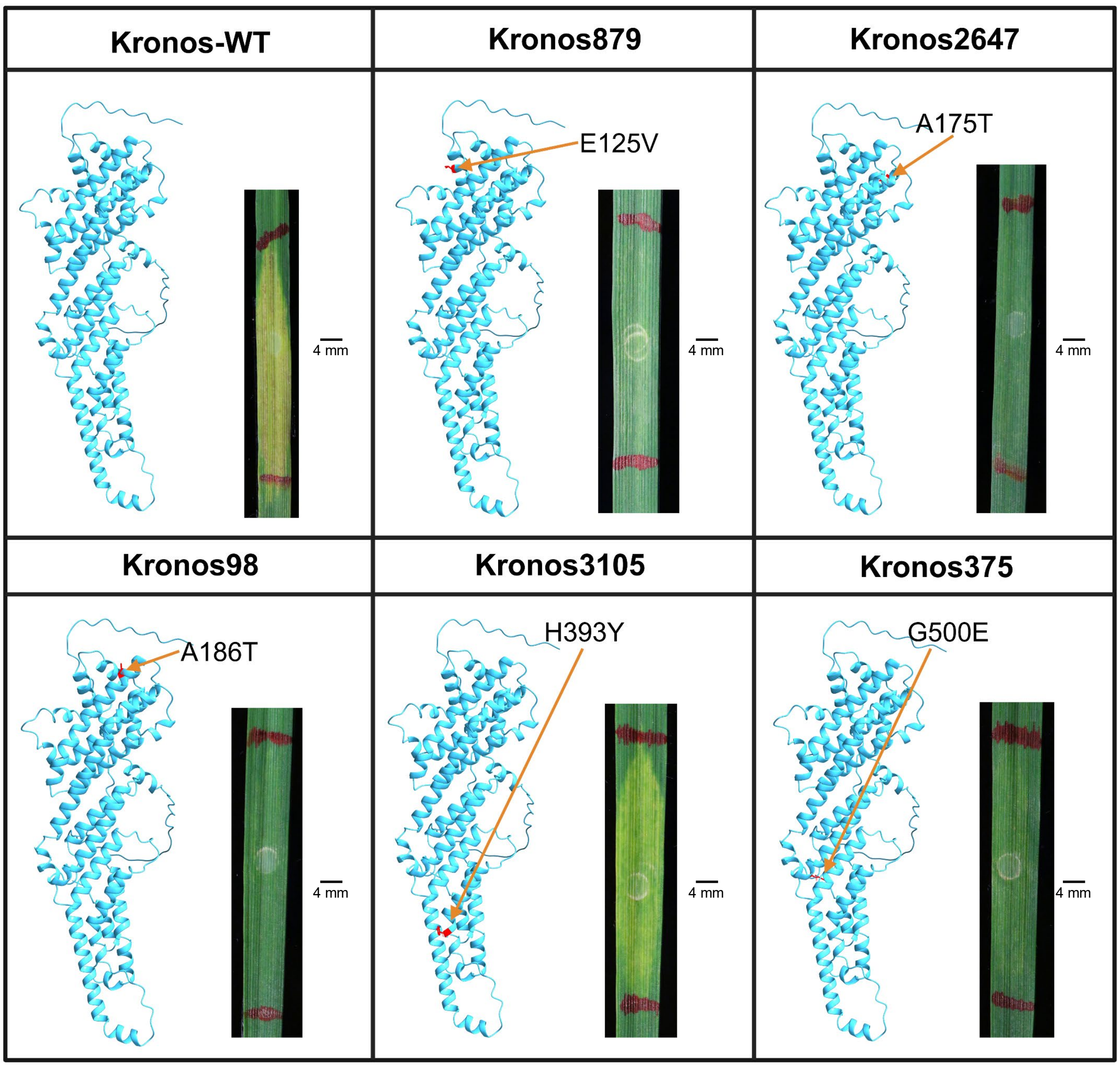

Supplementary Figure 2. Leaf infiltrations and TtExo70FX15 (Tsc2) protein structure visualizations for Prosper wild type (WT) and eleven EMS-induced ToxB-insensitive missense mutants identified in Prosper. Protein structures depict the AlphaFold-predicted wild-type TtExo70FX15 (Tsc2) protein, with missense mutation sites highlighted to indicate their spatial positions. The locations of the point mutations are shown in red and labeled next to orange arrows. Representative ToxB infiltration reactions for each mutant are shown on the right. Plants were infiltrated with ToxB at least three times. (Continued on next page)

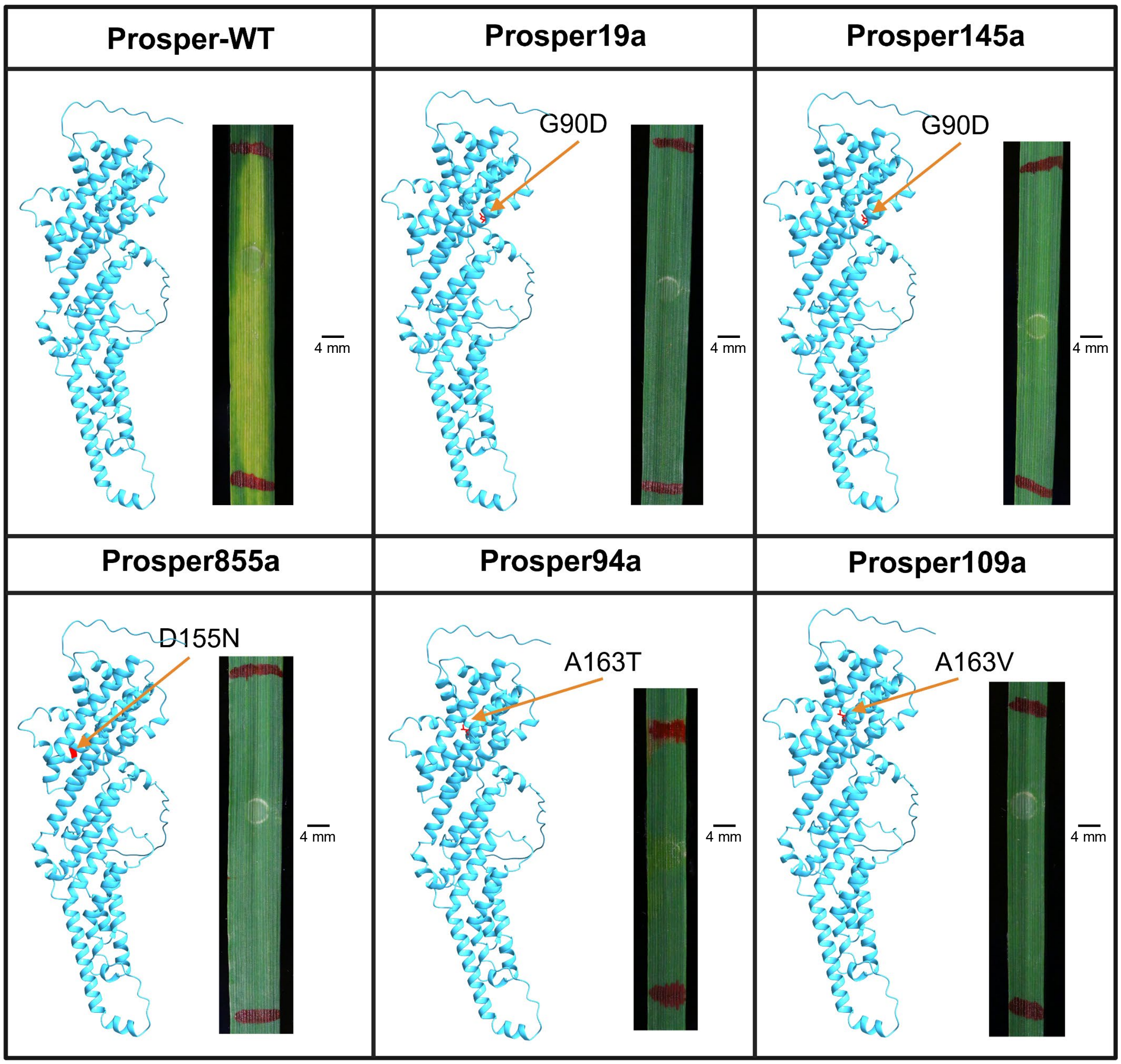

Supplementary Figure 2 (continued from previous page).

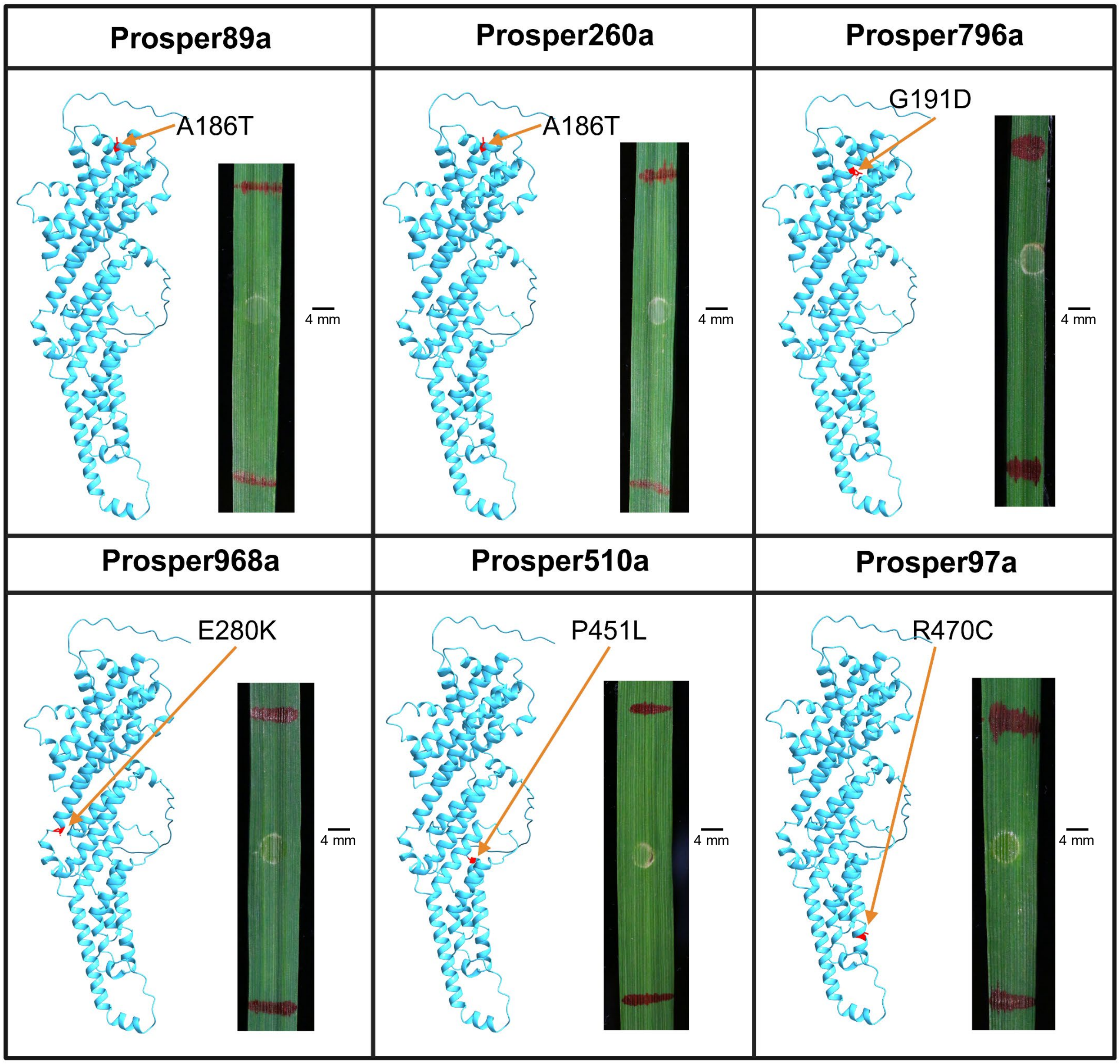

Supplementary Figure 3. Yeast two-hybrid (Y2H) assay to test the interaction between Tsc2 and ToxB. Yeast co-transformants carrying pGBKT7-ToxB and pGADT7-Tsc2 grew on SD/-Leu/-Trp (DDO), confirming successful co-transformation. However, no growth was observed on QDO/X-α-gal, indicating that the downstream reporter genes were not activated, and no direct interaction occurred. The positive control (pGBKT7-53 × pGADT7-T) showed strong growth on both DDO and QDO/X-α-gal, leading to blue coloration, while the negative control (pGBKT7-Lam × pGADT7-T) grew on DDO but failed to grow on QDO/X-α-gal.

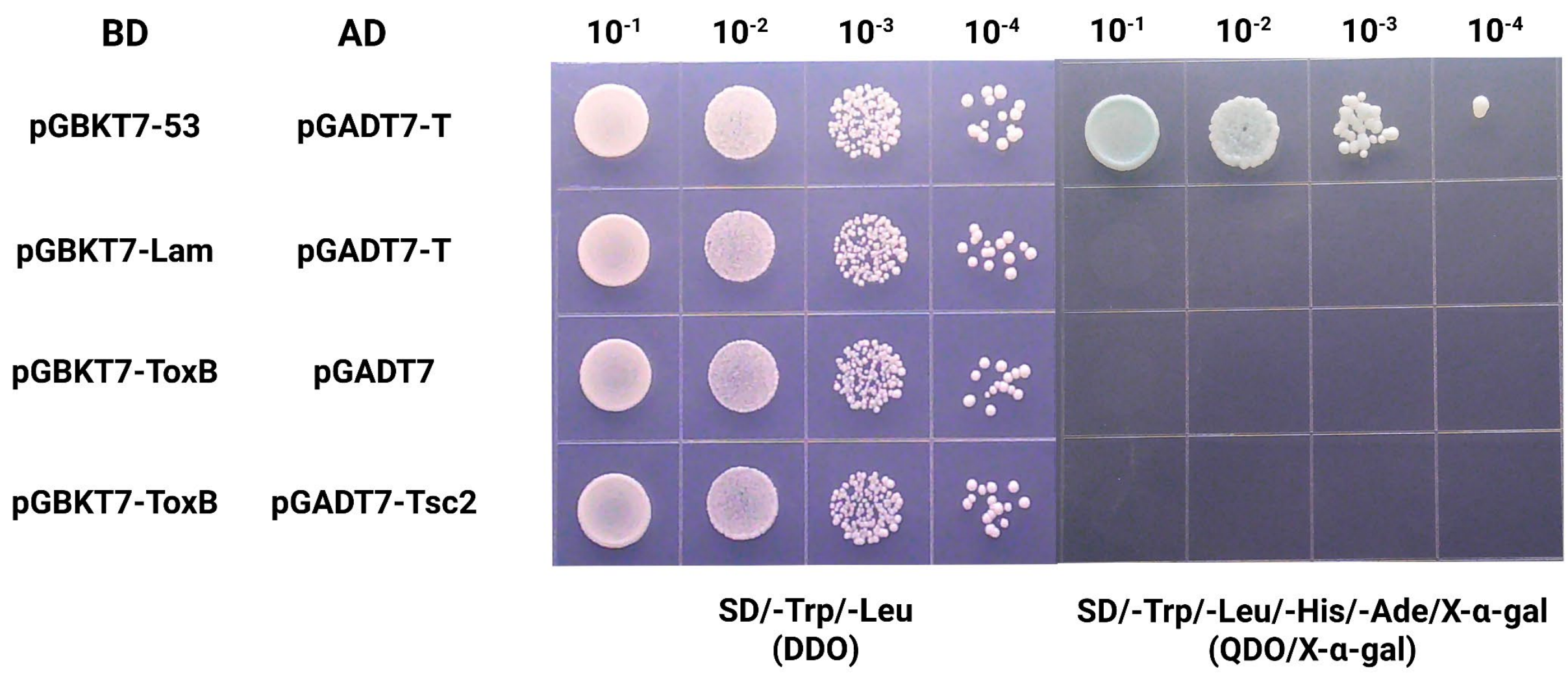

Supplementary Figure 4. Nuclear fluorescence intensity gradient analysis of GFP+NLS (nuclear localization signal protein; NLS) (**a**) and five independent gradient measurements of the Tsc2 protein::GFP + NLS (TSC2+NLS) (**b-f**).

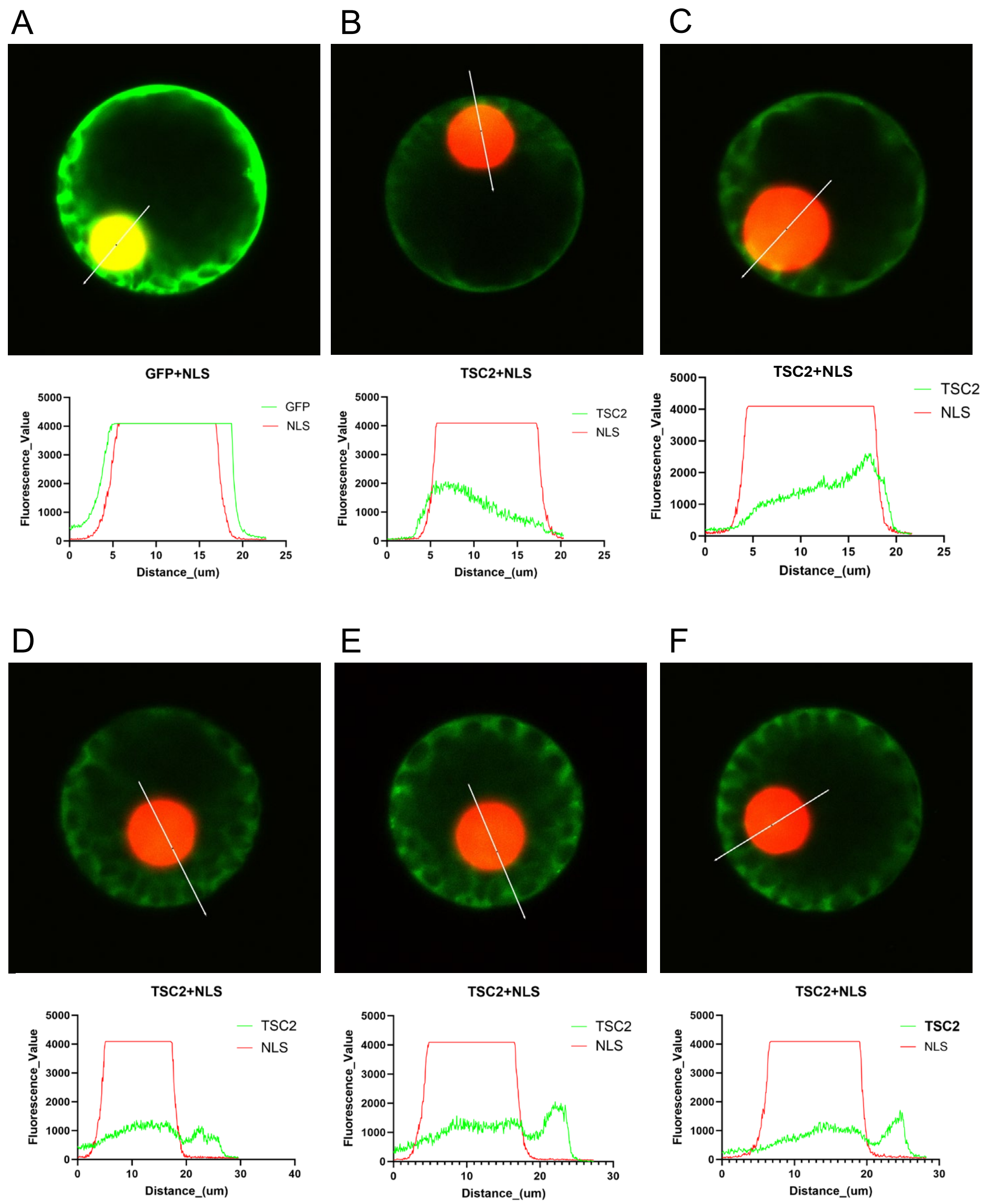

Supplement: Supplementary file 1 [file 41467_2026_77720_MOESM1_ESM.pdf]
