# Supplementary material for: The hijacking of a wheat Exo70-like protein by a fungal pathogen triggers disease
Source: Nat Commun. 2026 Sep 10;17:10713. doi: 10.1038/s41467-026-77720-9 (PMC13658248; doi:10.1038/s41467-026-77720-9)
Supplement: Supplementary file 2 [file 41467_2026_77720_MOESM2_ESM.pdf]

Description of Additional Supplementary Files

Supplementary Data 1: 2,037 single-nucleotide polymorphism (SNP) markers from the Illumina iSelect 90K SNP array used to assemble linkage groups corresponding to 14 chromosomes in the biparental population consisting of 149 recombinant inbred lines derived from the durum cultivars Kronos and Gredho.

Supplementary Data 2: Phenotypic reactions of 149 recombinant inbred lines derived from the durum cultivars Kronos and Gredho and checks in response to infiltration with the nectrophic effector ToxB.

Supplementary Data 3: PCR primers developed and used for saturation mapping of the *Tsc2* gene in the Kronos x Gredho recombinant inbred population.

Supplementary Data 4: Annotated gene models in the 108.4 kb candidate region of the Kronos genome defined by flanking recombination events identified through high-resolution mapping.

Supplementary Data 5: ToxB reactions and candidate gene analysis comparing the six candidate genes identified in Kronos across 12 sequenced wheat lines.

Supplementary Data 6: Kronos TILLING lines with mutations in the LRR-RLK gene *TrturKRN2B01G009130* and their reaction to infiltration with ToxB.

Supplementary Data 7: Kronos TILLING and Prosper EMS lines with mutations in the *TtExo70FX15* (*Tsc2*) gene, their reaction to ToxB, and their predicted impact on protein integrity.

Supplementary Data 8: Raw quantification cycle (Cq) value underlying *Tsc2* expression analyses across treatments and timepoints.

Supplementary Data 9: Summary of *Tsc2* transcriptional responses to ToxB treatment across different time points.

Supplementary Data 10: Presence/absence of *Tsc2* and reaction to ToxB in 112 hexaploid spring wheat lines.

Supplementary Data 11: Presence/absence of *Tsc2* and reaction to ToxB in 58 hexaploid winter wheat lines.

Supplementary Data 12: Presence/absence of *Tsc2* and reaction to ToxB in 99 durum wheat lines.

Supplementary Data 13: Presence/absence of *Tsc2* and reaction to ToxB in 121 accessions of tetraploid wheat relatives of durum.

Supplementary Data 14: *Tsc2* haplotypes and reaction to ToxB infiltrations of 177 Triticum accessions, including common wheat (spring and winter types), durum wheat, cultivated emmer, wild emmer, and Persian wheat.

Supplementary Data 15: Comparison of predicted binding-cavity druggability metrics for Exo70FX15 proteins from Kronos (*Triticum turgidum* ssp. *durum*), which carries the 21-nt deletion, and B-16 (*T. turgidum* ssp. *diccocoides*), which retains the 21-nt region.

Supplementary Data 16: Durum lines (n = 470) from the Global Durum Panel (GDP) evaluated for reaction to ToxB and genotyped with FAASA assays *fcp1066* and *fcp1077*.

Supplementary Data 17: Hexaploid winter wheat lines (n = 235) evaluated for reaction to ToxB and genotyped with FAASA assay *fcp1066*.

Supplementary Data 18: Hexaploid spring wheat lines (n = 778) evaluated for reaction to ToxB and genotyped with the FAASA assay *fcp1066*.

Supplementary Data 19: Summary of CRISPR/Cas9 transformation, editing recovery, and T_1 genotype-phenotype segregation for *Tsc2* in Kronos.

Supplementary Data 20: PCR primers used for analysis of the *Tsc2* gene for various purposes.
