# Supplementary material for: The hijacking of a wheat Exo70-like protein by a fungal pathogen triggers disease
Source: Nat Commun. 2026 Sep 10;17:10713. doi: 10.1038/s41467-026-77720-9 (PMC13658248; doi:10.1038/s41467-026-77720-9)
Supplement: Supplementary file 3 [file 41467_2026_77720_MOESM4_ESM.pdf]

nature portfolio

Corresponding author(s): Justin Faris

Last updated by author(s): Sep 1, 2026

Reporting Summary

Nature Portfolio wishes to improve the reproducibility of the work that we publish. This form provides structure for consistency and transparency in reporting. For further information on Nature Portfolio policies, see our Editorial Policies and the Editorial Policy Checklist.

Statistics

For all statistical analyses, confirm that the following items are present in the figure legend, table legend, main text, or Methods section.

n/a	Confirmed	
[ ]	[x]	The exact sample size (*n*) for each experimental group/condition, given as a discrete number and unit of measurement
[ ]	[x]	A statement on whether measurements were taken from distinct samples or whether the same sample was measured repeatedly
[ ]	[x]	The statistical test(s) used AND whether they are one- or two-sided<br>*Only common tests should be described solely by name; describe more complex techniques in the Methods section.*
[x]	[ ]	A description of all covariates tested
[ ]	[x]	A description of any assumptions or corrections, such as tests of normality and adjustment for multiple comparisons
[ ]	[x]	A full description of the statistical parameters including central tendency (e.g. means) or other basic estimates (e.g. regression coefficient) AND variation (e.g. standard deviation) or associated estimates of uncertainty (e.g. confidence intervals)
[ ]	[x]	For null hypothesis testing, the test statistic (e.g. F, t, r) with confidence intervals, effect sizes, degrees of freedom and P value noted<br>*Give P values as exact values whenever suitable.*
[x]	[ ]	For Bayesian analysis, information on the choice of priors and Markov chain Monte Carlo settings
[x]	[ ]	For hierarchical and complex designs, identification of the appropriate level for tests and full reporting of outcomes
[x]	[ ]	Estimates of effect sizes (e.g. Cohen's *d*, Pearson's *r*), indicating how they were calculated

Our web collection on statistics for biologists contains articles on many of the points above.

Software and code

Policy information about availability of computer code

Data collection	No custom software was used for data collection.
Data analysis	Data analysis was performed using GenomeStudio v2.0 for SNP genotype calling, MapDisto v2.1.7 for genetic linkage analysis, MAFFT/MAFFT-DASH v7.520 for multiple-sequence and structure-guided sequence alignment, and RAxML v8.2.12 for phylogenetic analyses. Comparative genomics and gene model prediction in unannotated assemblies were performed using AUGUSTUS (wheat parameters), with functional annotation supported by InterProScan v5.52-86.0 and the NCBI Conserved Domain Database. Marker development and primer design were conducted using Krait v1.3.3, Primer3, and PolyMarker. Protein structures were predicted using AlphaFold2 implemented via the ColabFold v1.5.2-patch pipeline, aligned using TM-align, and visualized using UCSF ChimeraX v1.6.1. Sequence assembly and haplotype analyses were conducted using Geneious Prime v2025.1.3. Quantitative PCR data were analyzed using Bio-Rad CFX Maestro v2.3, and statistical comparisons were performed in Python v3.12.13 using the 'scipy.stats.ttest_ind' module in SciPy v1.17.0. Online tools used for variant and structural impact prediction included SIFT, Missense3D, DynaMut2, and CavityPlus. Phylogenetic trees were visualized and annotated using iTOL v7.3. The predicted wild-type and CRISPR-derived Tsc2 amino acid sequences shown in Fig. 9c were visualized using WebLogo 3. No custom algorithms or software were developed.

For manuscripts utilizing custom algorithms or software that are central to the research but not yet described in published literature, software must be made available to editors and reviewers. We strongly encourage code deposition in a community repository (e.g. GitHub). See the Nature Portfolio guidelines for submitting code & software for further information.

Data

Policy information about availability of data

All manuscripts must include a data availability statement. This statement should provide the following information, where applicable:

- Accession codes, unique identifiers, or web links for publicly available datasets
- A description of any restrictions on data availability
- For clinical datasets or third party data, please ensure that the statement adheres to our policy

All data supporting the findings of this study are available within the article its Supplementary information and the accompanying Source Data files. The Tsc2 haplotype sequences generated from 177 accessions have been deposited in GenBank (https://www.ncbi.nlm.nih.gov/genbank/) under accession numbers PV543977-PV544011, PV544013-PV544024, PV544026-PV544057, PV544059-PV544080, PV544082-PV544091, PV544093-PV544116, PV544118-PV544138, and PV544140-PV544160. All genetic materials generated and used in this study are available from the corresponding author upon reasonable request and completion of a material transfer agreement (MTA) unless the materials were acquired elsewhere and not distributable by the corresponding author.

Research involving human participants, their data, or biological material

Policy information about studies with human participants or human data. See also policy information about sex, gender (identity/presentation), and sexual orientation and race, ethnicity and racism.

Reporting on sex and gender	Our study did NOT involved any human research participants.
Reporting on race, ethnicity, or other socially relevant groupings	Our study did NOT involved any human research participants.
Population characteristics	Our study did NOT involved any human research participants.
Recruitment	Our study did NOT involved any human research participants.
Ethics oversight	Our study did NOT involved any human research participants.

Note that full information on the approval of the study protocol must also be provided in the manuscript.

Field-specific reporting

Please select the one below that is the best fit for your research. If you are not sure, read the appropriate sections before making your selection.

[x] Life sciences [ ] Behavioural & social sciences [ ] Ecological, evolutionary & environmental sciences

For a reference copy of the document with all sections, see nature.com/documents/nr-reporting-summary-flat.pdf

Life sciences study design

All studies must disclose on these points even when the disclosure is negative.

Sample size	Sample sizes were determined based on established practices in wheat genetics and plant pathology. Large mapping populations were used to ensure sufficient recombination for high-resolution genetic mapping. No statistical methods were used to predetermine sample size.
Data exclusions	No data were excluded from the analyses.
Replication	All key experiments were repeated independently with similar results. Phenotyping experiments were conducted on multiple plants per genotype, and gene expression analyses included biological and technical replicates as described in the Methods.
Randomization	Randomization was not applicable, as plants were assigned to experimental groups based on genotype. For the sampling in the study, we always randomly select individuals within the group.
Blinding	Blinding was not applicable, as phenotypic outcomes were visually distinct and dependent on known genotypes. Investigators were not blinded because these kinds of experiments did not genetically need blinding.

Reporting for specific materials, systems and methods

We require information from authors about some types of materials, experimental systems and methods used in many studies. Here, indicate whether each material, system or method listed is relevant to your study. If you are not sure if a list item applies to your research, read the appropriate section before selecting a response.

Materials & experimental systems

n/a	Involved in the study	
☒	☐	Antibodies
☒	☐	Eukaryotic cell lines
☒	☐	Palaeontology and archaeology
☒	☐	Animals and other organisms
☒	☐	Clinical data
☒	☐	Dual use research of concern
☐	☒	Plants

Methods

n/a	Involved in the study	
☒	☐	ChIP-seq
☒	☐	Flow cytometry
☒	☐	MRI-based neuroimaging

Plants

Seed stocks	Sources and availability of all wheat seed stocks are described in the Methods.
Novel plant genotypes	Novel wheat genotypes generated in this study include EMS-induced mutants, CRISPR/Cas9-edited lines, and transgenic complementation lines, as described in the Methods.
Authentication	Plant genotypes were authenticated using gene-specific PCR markers, sequencing, and phenotypic assays as described in the Methods.
