# Supplementary material for: The hijacking of a wheat Exo70-like protein by a fungal pathogen triggers disease
Source: Nat Commun. 2026 Sep 10;17:10713. doi: 10.1038/s41467-026-77720-9 (PMC13658248; doi:10.1038/s41467-026-77720-9)
Supplement: Supplementary file 4 [file 41467_2026_77720_MOESM5_ESM.pdf]

nature portfolio

Peer Review File

The hijacking of a wheat Exo70-like protein by a fungal pathogen triggers disease

Corresponding Author: Dr Justin Faris

This file contains all reviewer reports in order by version, followed by all author rebuttals in order by version.

Version 0:

Reviewer comments:

Reviewer #1

(Remarks to the Author)
This manuscript reports the positional cloning, functional characterization, and evolutionary analysis of the wheat tan spot susceptibility gene Tsc2 (encoding Exo70FX15), and develops practical molecular markers and CRISPR/Cas9 editing target for breeding resistant varieties. The study is scientifically rigorous, innovative, and of high practical value. This is the first study to identify an Exo70 family protein as a susceptibility gene for necrotrophic fungal effectors, expanding the paradigm of plant-necrotroph interactions beyond typical receptor-like genes. The experimental design is complete and convincing, taking positional cloning, mutant validation, transgenic complementation, and genome editing. Also, the developed breeding tools fill a gap for efficient tan spot resistance improvement in wheat.
The manuscript is well-structured, with clear logic between results and discussion, and high-quality figures/tables that effectively support the core conclusions.
Comments and suggestions for improving the current manuscript:
1. The barley Exo70FX12 is required for no host resistance of wheat stripe rust locus Rps8, while wheat Exo70FX15 acts as a susceptibility gene for tan spot. While the Exo70FX12 is required for the genetic linked receptor kinase (Pur1) -mediated immunity in barley, it seems the receptor kinase protein encoding gene (LRR-RLK; TrturKRN2B01G009130) has no impact on the function of genetic-linked Exo70FX15 for tan spot. Is this difference related to the pathogenicity divergence of biotrophic and necrotrophic pathosystem? This striking functional divergence in the same Exo70FX clade should be emphasized in the introduction, results and discussion. The functional plasticity of Exo70FX proteins in Triticeae could further highlight the novelty of this study and its significance for understanding Exo70 evolution.
2. The inverse gene-for-gene concept is introduced multiple times. It can be clearly defined once in the introduction to link Ptr NEs to host susceptibility genes and only briefly referenced in subsequent sections to improve readability.
3. For CRISPR/Cas9 editing, the editing efficiency of T0 plants should be provided.
4. For the yeast two-hybrid (Y2H) assay, only full-length Tsc2 was tested and truncated Tsc2 variants were not assayed to rule out the possibility of direct interaction blocked by spatial hindrance of the full-length protein.
5. For Ptr ToxB infiltration, add the scoring standard for sensitivity/insensitivity (e.g., 0 = no chlorosis, 1 = mild chlorosis, 2 = severe chlorosis/cell death) to make the phenotypic data more reproducible for other researchers.
6. For the haplotype analysis, could the authors add a geographic distribution map/table of the five Tsc2 haplotypes to analyze the distribution pattern of susceptible/insensitive alleles in different regions and link it to breeding history and agricultural practices (e.g., conservation tillage)? Are the haplotype distribution in Table 1 showed significant difference in haplotype frequency among different wheat classes (common, durum, emmer)? This may highlight the unique enrichment of Haplotype 1 in cultivated durum/common wheat.
7. The manuscript notes that Tsc2-absent alleles are common in nature. The evolutionary driving force of the 21-nt deletion should be discussed. The manuscript hypothesizes the deletion arose in durum wheat and spread via breeding; further discuss whether the deletion is linked to beneficial agronomic traits (e.g., stem rust resistance in lumillo/Thatcher) and maintained by linkage disequilibrium—this explains why the susceptible allele was retained in breeding despite tan spot pressure. Add a discussion on whether Tsc2 has any physiological functon(e.g., growth, development, abiotic stress resistance) and whether its disruption causes any pleiotropic effects (based on phenotypic observations of edited/mutant lines) to reassure breeders of the safety of using Tsc2-knockout lines.
8. The current genome editing validation is limited to indoor Ptr ToxB infiltration. Are the T1 homozygous edited lines has any adverse effects on agronomic traits when Tsc2 disruption confers resistance under natural condition?
9. Based on the nucleocytoplasmic localization and no direct interaction with Ptr ToxB, propose a more specific indirect recognition pathway hypothesis (e.g., Ptr ToxB is recognized by a plasma membrane RLK, which recruits Tsc2 via an

unknown adaptor protein to regulate nuclear transcription/ cytoplasmic PCD signaling).
10. Expand the comparison with other tan spot susceptibility genes: Compare Tsc2 (Exo70FX15) with Tsn1 (NLR) and Tsc1 (LRR-RLK) in terms of protein structure, subcellular localization, and effector recognition mechanism to construct a comprehensive susceptibility network for Ptr ToxA/ToxB/ToxC in wheat.
11. Fig. 1, Fig. 4A and Fig. 7A (phenotype): Add a scale bar to the leaf images to quantify the size of chlorotic lesions and make the phenotypic difference more intuitive.
12. Fig. 2D (physical map): Annotate the putative functions of all six predicted genes in the 108.4 kb interval (not just the two candidates) to show the rigor of candidate gene screening.
13. Fig. 6A (phylogenetic tree): Add Exo70FX15 sequences from other major gramineous crops (e.g., rice, maize, sorghum) to expand the evolutionary analysis and clarify the conservation/divergence of Exo70FX15 in Poales.
14. Supplementary Figs.1-2 (protein structure): Unify the scale bar and color scheme for all mutant protein structures to facilitate direct comparison of mutation site positions.

Reviewer #2

(Remarks to the Author)
I have reviewed the research manuscript draft entitled “The hijacking of a wheat Exo70-like protein by a fungal pathogen triggers disease” by Singh and colleagues that was submitted to Nature Comms (NCOMMS-26-009161). The paper describes the cloning and partial characterisation of Tsc2 from the fungal pathogen Pyrenophora tritici-repentis. Tsc2 encode a putative Golgi component that is associated with other proteins involved with secretion. The study identified a genetic haplotype lacking 21 nucleotides conferring sensitivity to the ToxB effector and resulting in the onset of Tan spot of wheat. The study did a thorough validation of the newly discovered Tsc2 isoform, as well as developed markers that can be used for crop improvements. My comments and suggestions are below.

Page 9 and Figure 5B. ToxB suppression of Tsc2 expression either through the 1. onset of cell death and/or 2. feedback regulation. Does it also suppress the expression of ToxB-insensitive Tsc2? Furthermore, does biologically relevant infection with Ptr carrying ToxB only (race 5 I think?) suppress Tsc2 in the same way as protein infiltration alone? There are a few questions here that were not explored. Please consider this as I feel that further exploration into this area with basic RT-PCR will yield some useful knowledge that will add to the strength of this paper and Figure 5. Also, I suggest using a line graph for time series experiments.

Page 10, line 206-210 and Figure 6C. There is some ambiguity regarding Tsc2 localisation. Looks to me like GFP for both GFP only and Tsc2-GFP localised where chlorophyll/chloroplasts clustered in the two cells shown. Perhaps include images where the two cells are similarly orientated and perform a fluorescent intensity gradient analysis for proper subcellular quantification of fluorescence intensity. Also, please include DAPI staining to validate nuclear localisation.

Page 12, line 237. I am not sure if ‘gain-of-function’ is the best term to describe this. Doesn’t seem to improve its biological function or fitness in the host (wheat) organism by losing these 21 nt. Did you notice or determine if +21nt Tsc2 vs –21nt Tsc2 vs tsc2 varieties possess differences yield or phenotypic traits? Perhaps tone it down to “rendering it sensitive to PtrToxB”?
Page 16, bottom paragraph. This is interesting. The discussion focuses on the movement and origin of the Tsc2 hap 1. The study indicated that the insensitive variant is the primitive form giving rise to the ToxB sensitive variant. Thus, ToxB-Tsc2hap1 is a chicken-egg scenario. Ie which one comes first. I am curious to hear the authors thoughts/hypothesis on how ToxB comes about. Do you think or have clues whether ToxB have another unknown function in Ptr before Tsc2 loses the 21 nt?

Reviewer #3

(Remarks to the Author)
I will recommend acceptance as is, although I listed minor suggestion. This is very impactful and through piece of work, and sure will have significant impact on our knowledge and screening for resistance to the pathogen. It is very comprehensive approach to answer a 40 years old standing question, what is the gene coding for Tsc2? not only it answer to that, but it also open whole new area in the interaction mechanisms used in plant-pathogen interactions, expanding on known classes of sensitivity genes. added to the scientific advancement, there is a huge impact to be seen on the agriculture industry/scientists by identifying Tsc2/Exo70 as the susceptibility gene for a major necrotrophic effector in a major fungal disease of wheat. The work was extensive and is a classical example of work that one can teach in grad student courses these days. the authors went into all steps from purification of the ToxB effector to screening mapping population , followed by genetic linkage analysis, then fine mapping to narrow down the region from 5.6 cM to reach 0.1cM, enabled by screening with markers, phenotyping thousands of lines. followed by validation and forward genetic screen EMS-treated M2 families. followed by gain of function, transgenic complementation to confirm the role of Exo70 with no doubt and detailed the role 21nt indel in its function, defining several haplotypes so far. sure this work likely took several years of concentrated efforts!
I have read this manuscript over and over multiple times, but I only have very minor comments here and the authors can feel free to take it or ignore it. in the introduction, I think we have now agreed on the revised nomination of these toxins in Ptr, so page4, line 59, maybe just use ToxA, ToxB instead of Ptr ToxA and Ptr ToxB. The corresponding author was on the revised nomenclature work released few years ago as per the link https://doi.org/10.1094/PHYTO-01-23-0017-SC . And the other minor comments in the discussion on page 17 line 363, while the authors speak of 4-9% of sensitivity to ToxB in winter wheat, however in other work screening winter wheat lines from Europe showed 20% susceptibility to R5 (ToxB) isolate,

while in Canadian durum wheat that percentage was about 6-7%, however in durum wheat it was over 30%. find the info in this reference DOI: 10.1080/07060661.2020.1870002 .the images are of great quality showing all the details of the work!

Reviewer #4

(Remarks to the Author)
This manuscript by Singh et al., reports the cloning and functional validation of wheat Tsc2 gene, the wheat tan spot susceptibility gene, which encodes a Exo70FX15. The authors conducted genetic mapping, fine mapping, mutagenesis, transgenic complementation, and allelic diversity analysis to convincingly identify Tsc2 and demonstrate that its elimination through reading or gene editing results in enhanced resistance. It is interesting that a 21-nt deletion that spread through breeding provides important evolutionary and agronomic context. The study is clearly written, logically organized, and outlines and results presented from gene discovery to application.

The strength is the authors present Compelling genetic evidence based on positional cloning strategy that is rigorous, and complementation data strongly support gene identity. The genetic framework is the strongest aspect of the study. In addition, the breeding relevance is strong and relevant for crop improvement. Demonstrating marker-assisted breeding and gene editing as viable routes to improved resistance significantly enhances impact. The identification and historical tracing of the 21-nt deletion add depth and highlight unintended consequences of breeding.

The big weakness I observe includes very Limited mechanistic depth. While gene identification is convincing, the biochemical and mechanistic characterization remains relatively shallow. The conclusion that Tsc2 does not interact directly with the effector is based largely on Y2H assays, which are not definitive. In planta interaction assays such as co-IP would strengthen this claim.

Regardless, this is a strong genetic study with clear application and solid data supporting Tsc2 identification. However, the mechanistic advance is modest relative to the strength of the genetic findings. Strengthening interaction assays or expanding the mechanistic framework would elevate the manuscript’s impact.
The work is publishable and valuable, but additional mechanistic clarity would significantly enhance its contribution.

Version 1:

Reviewer comments:

Reviewer #1

(Remarks to the Author)
The revised manuscript made significant revision based on the comments of the reviewers. The authors performed additional experiments and provided new information to support their findings. I was satisfied with the scientific meaning and content of the revision. I have no further comments for the revised manuscript.

Reviewer #2

(Remarks to the Author)
I've had the opportunity to evaluate the revised manuscript and do not have additional comments. Faris and his team have provided a very nice and insightful rebuttal. Thank you!

Open Access This Peer Review File is licensed under a Creative Commons Attribution 4.0 International License, which permits use, sharing, adaptation, distribution and reproduction in any medium or format, as long as you give appropriate credit to the original author(s) and the source, provide a link to the Creative Commons license, and indicate if changes were made.
In cases where reviewers are anonymous, credit should be given to 'Anonymous Referee' and the source.
The images or other third party material in this Peer Review File are included in the article’s Creative Commons license, unless indicated otherwise in a credit line to the material. If material is not included in the article’s Creative Commons license and your intended use is not permitted by statutory regulation or exceeds the permitted use, you will need to obtain permission directly from the copyright holder.
To view a copy of this license, visit https://creativecommons.org/licenses/by/4.0/

Reviewer #1 (Remarks to the Author)

This manuscript reports the positional cloning, functional characterization, and evolutionary analysis of the wheat tan spot susceptibility gene Tsc2 (encoding Exo70FX15), and develops practical molecular markers and CRISPR/Cas9 editing target for breeding resistant varieties. The study is scientifically rigorous, innovative, and of high practical value. This is the first study to identify an Exo70 family protein as a susceptibility gene for necrotrophic fungal effectors, expanding the paradigm of plant-necrotroph interactions beyond typical receptor-like genes. The experimental design is complete and convincing, taking positional cloning, mutant validation, transgenic complementation, and genome editing. Also, the developed breeding tools fill a gap for efficient tan spot resistance improvement in wheat.

The manuscript is well-structured, with clear logic between results and discussion, and high-quality figures/tables that effectively support the core conclusions.

Comments and suggestions for improving the current manuscript:

1. The barley Exo70FX12 is required for no host resistance of wheat stripe rust locus Rps8, while wheat Exo70FX15 acts as a susceptibility gene for tan spot. While the Exo70FX12 is required for the genetic linked receptor kinase (Pur1) -mediated immunity in barley, it seems the receptor kinase protein encoding gene (LRR-RLK; TrturKRN2B01G009130) has no impact on the function of genetic-linked Exo70FX15 for tan spot. Is this difference related to the pathogenicity divergence of biotrophic and necrotrophic pathosystem? This striking functional divergence in the same Exo70FX clade should be emphasized in the introduction, results and discussion. The functional plasticity of Exo70FX proteins in Triticeae could further highlight the novelty of this study and its significance for understanding Exo70 evolution.

Response: This is a great question, and it gets to the crux of this research. First, it should be noted that barley Exo70FX12 and wheat Exo70FX15 are not orthologous, and barley Pur1 and wheat TrturKRN2B01G009130 are not orthologous. Wheat has orthologs of barley Exo70FX12 and Pur1 on chromosome 4B. However, because the closely related barley Exo70FX12 gene relies on the presence of Pur1 to confer resistance, we feel it is possible that Tsc2 (Exo70FX15) also requires a functional LRR-RLK to confer susceptibility. But we ruled out TrturKRN2B01G009130 as the LRR-RLK involved. Our EMS mutagenesis experiments led only to the identification of the Exo70FX15 gene and never a different locus (e.g., an LRR-RLK). Therefore, our hypothesis for this is that there is likely genetic redundancy for the LRR-RLK gene associated with the Exo70FX15, which is likely because wheat is polyploid, and therefore, if one of them is mutated, others will compensate for that, which makes it difficult to identify. The functions of Exo70FX12 in barley and Exo70FX15 in wheat are

most likely the same (i.e., induction of PCD, defense response, etc.), but the difference in pathogen biology between biotrophs and necrotrophs, where biotrophs (stripe rust fungus) are thwarted by such a response and necrotrophs take advantage and thrive, leads to resistance in the former and susceptibility in the latter. Therefore, it is not technically functional divergence in the host gene, but amongst fungal pathogens. We have made several edits in the introduction (last paragraph) and discussion to better emphasize this point.

2. The inverse gene-for-gene concept is introduced multiple times. It can be clearly defined once in the introduction to link Ptr NEs to host susceptibility genes and only briefly referenced in subsequent sections to improve readability.

Response: Thank you for this comment. ‘Inverse gene-for-gene’ occurs once in the abstract and once in the introduction.

3. For CRISPR/Cas9 editing, the editing efficiency of T0 plants should be provided.

Response: We have now added these details to the Results (Lines 319-327) and Methods (Lines 711-725) sections and have included a new Supplementary Table 19 summarizing the T0 transformation, transgene recovery, editing outcome, and T1 genotype-phenotype results.

Briefly, 100 Kronos immature embryos were inoculated/co-cultivated with Agrobacterium carrying the JD633-CRISPR/Cas9-Tsc2 construct. This yielded two regenerated T0 plants derived from independent calli. PCR screening identified one transgene-positive T0 plant, which was sequenced at the target locus and found to carry the single-base insertion g.39_40insC. Thus, one edited T0 plant was recovered from 100 inoculated embryos and from two regenerated T0 plants; the single transgene-positive T0 plant analyzed at the target locus carried the edit. Because only one transgene-positive T0 plant was recovered, we now report the raw counts explicitly rather than emphasizing a percentage-based editing efficiency.

The edited T0 plant was self-pollinated, and five T1 progeny were genotyped and phenotyped. Two T1 plants carrying the edited g.39_40insC allele were insensitive to ToxB, whereas three plants carrying the wild-type Tsc2 target-site sequence remained sensitive. These results are now summarized in Supplementary Table 19.

4. For the yeast two-hybrid (Y2H) assay, only full-length Tsc2 was tested and truncated Tsc2 variants were not assayed to rule out the possibility of direct interaction blocked by spatial hindrance of the full-length protein.

Response: Yes, we tested only the full-length protein. We debated whether to do Y2H at all given that it has been shown previously that ToxB stays in the apoplast (Figueroa et al. 2015) and our finding that Exo70FX15 does not exit the cell. We felt that additional Y2H experiments would not be a productive use of resources.

5. For Ptr ToxB infiltration, add the scoring standard for sensitivity/insensitivity (e.g., 0 = no chlorosis, 1 = mild chlorosis, 2 = severe chlorosis/cell death) to make the phenotypic data more reproducible for other researchers.

Response: The definition of the scoring scale has been added to the materials and methods under the section 'Plant growth and phenotyping' (lines 472-474).

6. For the haplotype analysis, could the authors add a geographic distribution map/table of the five Tsc2 haplotypes to analyze the distribution pattern of susceptible/insensitive alleles in different regions and link it to breeding history and agricultural practices (e.g., conservation tillage)? Are the haplotype distribution in Table 1 showed significant difference in haplotype frequency among different wheat classes (common, durum, emmer)? This may highlight the unique enrichment of Haplotype 1 in cultivated durum/common wheat.

Response: This is a logical suggestion, and it is one that we have taken a close look at (see appendix to comments). Because the H1 (susceptible) haplotype appears to have originated in durum wheat, it made sense to analyze the GDP from a geographic perspective to see if we could uncover some evidence for the origin of the H1 haplotype and its relationship to H3. However, the results were inconclusive. We feel that a significant limitation to the analysis is sample size and unequal representation from different geographic regions. Some countries are represented by one or two lines whereas others had over 70. As a result, we are not comfortable drawing conclusions or speculating on the geographic origin of H1 until we have data for a much larger set of lines with better representation. We feel that a much larger more expansive analysis of lines consisting of several thousand common wheat, durum, and cultivated and wild emmer accessions with known collection sites would be necessary to draw solid conclusions. The markers that we have developed in this study would be extremely useful for such an experiment because they can be used to first determine which lines harbor a Tsc2 allele and then distinguish H1 from the other haplotypes without the need for phenotyping with ToxB infiltrations. If the reviewer is interested in more details

regarding our analysis, we refer them to the appendix included at the end of this document.

7. The manuscript notes that Tsc2-absent alleles are common in nature. The evolutionary driving force of the 21-nt deletion should be discussed. The manuscript hypothesizes the deletion arose in durum wheat and spread via breeding; further discuss whether the deletion is linked to beneficial agronomic traits (e.g., stem rust resistance in Iumillo/Thatcher) and maintained by linkage disequilibrium—this explains why the susceptible allele was retained in breeding despite tan spot pressure. Add a discussion on whether Tsc2 has any physiological functon (e.g., growth, development, abiotic stress resistance) and whether its disruption causes any pleiotropic effects (based on phenotypic observations of edited/mutant lines) to reassure breeders of the safety of using Tsc2-knockout lines.

Response: Iumillo possesses the stem rust resistance gene Sr9g on chromosome 2B, the same chromosome as Tsc2. However, Sr9g is on the long arm whereas Tsc2 is on the short arm and they are independently linked. We can conclude that the reason Iumillo was crossed to a hexaploid was to introgress stem rust resistance conferred by Sr9g, but we don't have evidence that Tsc2 was maintained in hexaploid varieties due to stem rust resistance linkage. Furthermore, tan spot was not a relevant disease until after 1940, and race 5 is rare in North America. Therefore, it is most likely that Tsc2 was carried along by chance or linked to something else beneficial, but we don't have evidence for what that would be. Therefore, we cannot say Tsc2 was maintained despite tan spot pressure. It may be that Tsc2 was spread amongst common wheat varieties not so much because it was linked to something that was selected for, but because Thatcher was used in a lot of breeding programs to generate new varieties at the time, and because tan spot was not a relevant disease until after 1940, breeders were not aware of the presence of Tsc2. At this point, this is all speculation, and more research beyond the scope of the current manuscript is needed to gain a better understanding.

Regarding the potential of Tsc2 having an alternate positive function, we feel that is unlikely because many high-performing wheat and durum varieties possess the absent allele, and we observed no adverse traits while growing these varieties. Some of our mutants displayed deleterious traits, but none that were common among all Tsc2 mutants, which suggests that those traits were due to alternate mutations being carried elsewhere in their genomes. We address this idea briefly in lines 429-435.

8. The current genome editing validation is limited to indoor Ptr ToxB infiltration. Are the T1 homozygous edited lines has any adverse effects on agronomic traits when Tsc2 disruption confers resistance under natural condition?

Response: We do not know as we are not allowed to grow transgenic/gene edited lines outside of a BSL-2 greenhouse facility. However, natural Tsc2-disrupted mutants (Tsc2-alleles and absent alleles) are routinely grown under field conditions with no adverse agronomic traits. Indeed, as mentioned above, there are many high-performing commercially grown wheat cultivars that are lacking Tsc2 altogether or have Tsc2-alleles.

9. Based on the nucleocytoplasmic localization and no direct interaction with Ptr ToxB, propose a more specific indirect recognition pathway hypothesis (e.g., Ptr ToxB is recognized by a plasma membrane RLK, which recruits Tsc2 via an unknown adaptor protein to regulate nuclear transcription/ cytoplasmic PCD signaling).

Response: Please see page 18, lines 378-384 where more description of the potential pathway is provided. We are reluctant to add more speculation beyond this without empirical data.

10. Expand the comparison with other tan spot susceptibility genes: Compare Tsc2 (Exo70FX15) with Tsn1 (NLR) and Tsc1 (LRR-RLK) in terms of protein structure, subcellular localization, and effector recognition mechanism to construct a comprehensive susceptibility network for Ptr ToxA/ToxB/ToxC in wheat.

Response: More discussion has been included on page 16 (lines 335-352) to expand comparison of NE sensitivity genes and their proteins not only among the wheat-Ptr genes but also including the wheat-P. nodorum system, which is a highly similar system.

11. Fig. 1, Fig. 4A and Fig. 7A (phenotype): Add a scale bar to the leaf images to quantify the size of chlorotic lesions and make the phenotypic difference more intuitive.

Response: Scale bars have been added to Figures 1B, 4A, and 7A based on the 4 mm diameter of the syringe end, which leaves a visible mark on infiltrated leaves. We were unable to add the scale to Figure 1A because no such mark was included on leaves inoculated with conidia. However, the leaves are of very similar age and size to the infiltrated leaves in Fig. 1B, and therefore the scale bar provides a good estimate for 1A as well.

12. Fig. 2D (physical map): Annotate the putative functions of all six predicted genes in the 108.4 kb interval (not just the two candidates) to show the rigor of candidate gene screening.

Response: We have revised Fig. 2D to annotate the predicted functions of all six gene models within the 108.4-kb candidate interval. The figure and legend now indicate the three transferase-family genes, the plant invertase/pectin methylesterase inhibitor-like domain protein, the LRR-RLK candidate, and the Exo70 exocyst subunit-family gene, subsequently identified as Tsc2/TtExo70FX15. We also added a brief description of the six predicted functions in the Results text (Lines 116-118) and retained the full gene annotations in Supplementary Table 4.

13. Fig. 6A (phylogenetic tree): Add Exo70FX15 sequences from other major gramineous crops (e.g., rice, maize, sorghum) to expand the evolutionary analysis and clarify the conservation/divergence of Exo70FX15 in Poales.

Response: We agree that the conservation and divergence of Tsc2/Exo70FX15 outside the Triticeae should be clarified. In short, we did not include rice, maize, and sorghum in the phylogenetic tree because they do not possess orthologs of Exo70FX15/Tsc2, i.e., the ExoFX11-derived genes Exo70FX12 and Exo70FX15 are specific to the Triticeae. A more detailed response is provided below. In Holden et al. (2022), Exo70FX15 was defined as a clade sister to Exo70FX12 and was reported in the genera Aegilops, Triticum, and Hordeum. That study further concluded that Exo70FX12 and Exo70FX15 are recent innovations within the Triticeae, both derived from Exo70FX11. Thus, although rice, maize, and sorghum contain Exo70FX-family members, the available phylogenetic evidence does not support the presence of clear orthologs of the Triticeae Exo70FX15/Tsc2 clade in these non-Triticeae grasses. We also examined the rice locus LOC_Os01g67810.1 (Os01g0905200) specifically because the rice database annotation includes OsEXO70FX15 among its gene symbols. However, the cross-species phylogenetic nomenclature used in the Holden et al. (2022) dataset (https://github.com/matthewmoscou/Exo70; accessed on 5 August 2026) designates LOC_Os01g67810.1 as OsExo70FX6b, and this protein was not the highest-scoring rice sequence recovered in our BLASTP search. We therefore interpret the rice OsEXO70FX15 label as a difference between historical rice-specific and cross-species Exo70FX nomenclature, rather than evidence that this rice locus belongs to the Triticeae Exo70FX15 branch. When inspected in the context of the Holden et al. Exo70FX phylogenetic framework, rice, maize, and sorghum sequences are clearly divergent Exo70FX paralogs rather than Tsc2/Exo70FX15 orthologs. The full-length 500-amino-acid Tsc2 protein sequence (GenBank protein accession XRO44311.1; CDS accession PV543985.1; this study) was searched against the NCBI non-redundant protein sequence database (nr) using BLASTP (https://blast.ncbi.nlm.nih.gov/Blast.cgi?PROGRAM=blastp&PAGE_TYPE=BlastSearch&LINK_LOC=blasthome) on 5 August 2026. The search was taxonomically restricted to Oryza sativa (taxid: 4530), Zea mays (taxid: 4577), and Sorghum (taxid: 4557, including

S. bicolor). BLASTP was run with an E-value threshold of 1 × 10^{-5}, a maximum of 100 target sequences, word size 6, the BLOSUM62 substitution matrix, gap-opening and gap-extension costs of 11 and 1, respectively, and low-complexity filtering enabled.

For this reason, we did not add rice, maize, or sorghum sequences to Fig. 6A as "Exo70FX15" orthologs, because doing so would risk implying an orthologous relationship that is not supported by the available phylogenetic evidence. Instead, we have revised the Results (Lines 208-212) to clarify that Tsc2 belongs to a Triticeae-associated Exo70FX15 lineage and that representative non-Triticeae grasses contain divergent Exo70FX-family members but lack clear orthologs of the Triticeae Exo70FX15/Tsc2 clade. Below is a table for the reviewers' information regarding the most closely related genes present in rice, Sorghum, and maize, and their relationship to Tsc2.

Species	Highest-scoring BLASTP accession	Corresponding gene model	Exo70FX designation	Identity	Query coverage	E-value
Oryza sativa	XP_015638069.1	LOC_Os05g30640.1	OsExo70FX9d	29.66%	81%	1.10 × 10^{-41}
Zea mays	XP_008645347.1	GRMZM5G815077_P01	ZmExo70FX8	28.49%	64%	2.95 × 10^{-28}
Sorghum bicolor	KAG0516287.1	Closely corresponding curated gene model: Sobic.010G271600.1.p	SbExo70FX13c	31.32%	78%	2.77 × 10^{-39}

Note: KAG0516287.1 was the actual highest-scoring sorghum accession recovered by BLASTP. Sobic.010G271600.1.p is the closely corresponding curated S. bicolor protein model used to assign the SbExo70FX13c designation; these identifiers should not be treated as interchangeable accessions. The Exo70FX subgroup designations were not inferred from the BLAST descriptions alone. The corresponding rice, maize, and sorghum gene-model identifiers were cross-referenced against the Exo70_gene_definitions.txt file in the M. Moscou curated Exo70 repository (https://github.com/matthewmoscou/Exo70), accessed on 5 August 2026. This resource assigns LOC_Os05g30640.1, GRMZM5G815077_P01, and Sobic.010G271600.1.p to OsExo70FX9d, ZmExo70FX8, and SbExo70FX13c, respectively.

14. Supplementary Figs.1-2 (protein structure): Unify the scale bar and color scheme for all mutant protein structures to facilitate direct comparison of mutation site positions.

Response: We added scale bars to each of the leaf infiltration images. Unfortunately, we are unsure of the Reviewer's meaning regarding the unification of color schemes. In our files, all the protein structures are light blue in color with the mutated amino acid highlighted in red and its position indicated with an orange arrow. All protein structures are shown in the same orientation to make it easy to compare mutated amino acid positions among different mutants.

Reviewer #2 (Remarks to the Author)

I have reviewed the research manuscript draft entitled "The hijacking of a wheat Exo70-like protein by a fungal pathogen triggers disease" by Singh and colleagues that was submitted to Nature Comms (NCOMMS-26-009161). The paper describes the cloning and partial characterisation of Tsc2 from the fungal pathogen Pyrenophora tritici-repentis. Tsc2 encode a putative Golgi component that is associated with other proteins involved with secretion. The study identified a genetic haplotype lacking 21 nucleotides conferring sensitivity to the ToxB effector and resulting in the onset of Tan spot of wheat. The study did a thorough validation of the newly discovered Tsc2 isoform, as well as developed markers that can be used for crop improvements. My comments and suggestions are below.

Page 9 and Figure 5B. ToxB suppression of Tsc2 expression either through the 1. onset of cell death and/or 2. feedback regulation. Does it also suppress the expression of ToxB-insensitive Tsc2? Furthermore, does biologically relevant infection with Ptr carrying ToxB only (race 5 I think?) suppress Tsc2 in the same way as protein infiltration alone? There are a few questions here that were not explored. Please consider this as I feel that further exploration into this area with basic RT-PCR will yield some useful knowledge that will add to the strength of this paper and Figure 5. Also, I suggest using a line graph for time series experiments.

Response: We appreciate this comment and agree that there is always more that can be explored when it comes to gene regulation, expression, and control. First, regarding the evaluation of gene expression under spore inoculation, i.e., tan spot disease caused by race 5, we did not undertake this experiment. In my lab and in my colleague's lab (T. L. Friesen), we have attempted to quantify expression of other NE sensitivity genes under inoculation, and the results have always been highly inconsistent. The reason is likely because it is impossible to generate a consistent number of infection sites and/or lesion sizes on a leaf under spore inoculations, especially if comparing resistant to susceptible reactions. In our experience, quantifying gene expression under infiltration is much more accurate, at least for evaluation of the host gene-NE interaction directly,

because essentially every cell has been exposed to the NE making it reminiscent of a single cell expression analysis.

Although we didn't feel it prudent to conduct the experiment to look at Tsc2 expression under inoculation, we did conduct experiments to address the reviewer's first question (does ToxB suppress the expression of an insensitive Tsc2 allele?). In the first experiment that we conducted to address this, we compared Kronos (Tsc2+) to a ToxB-insensitive Kronos mutant that codes for a truncated protein. However, we were surprised to find that the mutant Kronos allele was never expressed. At first, this was a perplexing result, but then we learned about an RNA-surveillance pathway known as nonsense-mediated decay, which leads to reduced mRNA levels of transcript with early stop codons. This explained the phenomenon that we observed, but it required that we choose a different insensitive genotype and repeat the experiment once again.

For our next try, we choose a durum line (DWRC-1014) with the insensitive allele belonging to haplotype 3. The only difference between this allele and the sensitive allele is the 21 nt indel. For this experiment, we were forced to perform it under conditions that varied from the one in the initial version of the manuscript. First, the experiment was conducted by a different person (now third author, Z. Zhang) because first author G. Singh has since left for a new position. Second, we were forced to use a different growth chamber with different light type (LED vs fluorescent) and a different light/dark cycle. In the first experiment, plants were exposed to a 16:8 hr photoperiod, whereas in the new experiment, the growth chamber had a 12:12 light/dark cycle (we could not change the settings because others were using it at the same time and their experiments required 12:12). The photoperiod is highly relevant because so far, all NE sensitivity genes have been shown to be controlled by light/dark. However, analysis under a 12-hr photoperiod is acceptable, especially given that we have used a 12-hr photoperiod to characterize the other NE sensitivity genes that we have cloned.

The different growth conditions altered the gene expression results somewhat compared to the original experiment. First, it should be noted that plants must be moved into darkness for approximately 10 minutes to render them amenable to infiltration. For our timepoint 0 in this experiment, plants had been exposed to light for about an hour before then being placed under darkness for infiltration. The sudden move to darkness caused Tsc2 expression to be extremely high for timepoint 0. Therefore, we omitted timepoint 0 from the analysis and used 6 hpi as the first timepoint, which allowed gene expression to revert to 'normal' levels under light.

The biggest difference that we observed between the new experiment and the original was the lack of divergence in Tsc2 expression between ToxB and YPD treated plants at

48 and 72 hours. Instead, trends were rather consistent between ToxB and YPD treated plants for both Kronos (H1) and DWRC-1014 (H3), with the exception that Tsc2 expression in ToxB-treated plants was significantly higher than YPD-treated plants in Kronos (H1), and this difference was not observed in the incompatible interaction (DWRC-1014). This indicates that ToxB tends to induce expression of Tsc2 in a compatible interaction (H1) for a short time (at the 24 hour timepoint), but not in an incompatible interaction (H3). The observation that Tsc2 expression in YPD-treated plants tended to increase beyond 24 hours in the initial experimental conditions but stayed relatively constant (with slight decline) under the new conditions in both compatible and incompatible interactions would suggest that environmental conditions including photopheriod and light intensity likely play a role in gene expression, which is not a surprise at all given that all the NE sensitivity genes that we've evaluated to date are regulated by light.

We realize there are unanswered questions regarding the light regulation of Tsc2 expression. For the other NE genes we've cloned, we conducted qPCR experiments sampling plants every three hours over four days under a 12-hour photoperiod, and each time we observed an oscillating expression pattern that followed a 24 hour rhythm. We didn't do that for Tsc2 because those experiments are so technically demanding and expensive, and there is not a lot of practical relevance to the outcome.

Overall, we feel that our new qPCR experiments address the reviewer's concern by showing that a similar expression trend of Tsc2 occurs under both compatible and incompatible (H1 vs H3 haplotypes) interactions. The results indicate that ToxB causes a significant induction of Tsc2 expression at 24 hours post infiltration in a compatible interaction, and then it drops to levels that are not significantly different from the controls. This induction is not observed in the incompatible interaction. Biologically, this makes sense in that the recognition of the effector leads to a rapid, upregulated host response to fend off the pathogen. The downstream 'defense response' machinery would be quickly activated leading to PCD, which the pathogen then exploits.

As the reviewer requested, we replaced the bar graph with line graphs. We also made updates in the results (lines 183-198) and in the materials and methods (lines 591, 598, 599, 604, and 605).

Page 10, line 206-210 and Figure 6C. There is some ambiguity regarding Tsc2 localisation. Looks to me like GFP for both GFP only and Tsc2-GFP localised where chlorophyll/chloroplasts clustered in the two cells shown. Perhaps include images where the two cells are similarly orientated and perform a fluorescent intensity gradient

analysis for proper subcellular quantification of fluorescence intensity. Also, please include DAPI staining to validate nuclear localisation.

Response: We acknowledge these concerns and therefore repeated the localization experiments (outsourced to Lifeasible). The experiment was performed as a co-localization assay in wheat mesophyll protoplasts using Tsc2-GFP together with the nuclear marker NLS-mKATE. We have now revised Fig. 6C to display the GFP, NLS-mKATE, chlorophyll autofluorescence, bright-field, and merged channels for both Tsc2-GFP and the GFP-only control. We also selected representative cells that are more directly comparable in orientation.

To further support the localization interpretation, we added a new Supplementary Fig. 4 containing fluorescence intensity profiles for the GFP-only control and five independent Tsc2-GFP-transformed protoplasts. These profiles show that the Tsc2-GFP signal overlaps the NLS-mKATE-defined nuclear region and is also detectable in the surrounding cytoplasm, supporting localization of Tsc2-GFP in both the nucleus and cytoplasm. We have also revised the Results (Lines 221-232) and Methods (Lines 642-653) to describe the co-expression of Tsc2-GFP with NLS-mKATE and the fluorescence intensity profile analysis. In the Discussion (Lines 377-380), we have minimally revised the wording to state that Tsc2-GFP localizes to the nucleus and cytoplasm rather than the plasma membrane, consistent with a role outside the canonical Exo70-associated exocyst-tethering pathway.

Page 12, line 237. I am not sure if 'gain-of-function' is the best term to describe this. Doesn't seem to improve its biological function or fitness in the host (wheat) organism by losing these 21 nt. Did you notice or determine if +21nt Tsc2 vs –21nt Tsc2 vs tsc2 varieties possess differences yield or phenotypic traits? Perhaps tone it down to "rendering it sensitive to PtrToxB"?

Response: We concur with this comment and have modified the statement to remove 'gain of function.' We receive the question about whether Tsc2 mutants might have other phenotypic effects quite often (see response to Reviewer 1 comments also). We have evaluated many mutants, both natural and induced, and have never observed another trait being affected that was consistent across mutants (sometimes a specific EMS-induced mutant might show a different trait phenotype, but presumably due to a mutation in another gene(s) because the mutation load is very high in EMS mutants). However, we cannot rule out the possibility that Tsc2 is influencing another trait for which we have not evaluated, such as response to a specific pathogen or abiotic stress.

Page 16, bottom paragraph. This is interesting. The discussion focuses on the movement and origin of the Tsc2 hap 1. The study indicated that the insensitive variant is the primitive form giving rise to the ToxB sensitive variant. Thus, ToxB-Tsc2hap1 is a chicken-egg scenario. Ie which one comes first. I am curious to hear the authors thoughts/hypothesis on how ToxB comes about. Do you think or have clues whether ToxB have another unknown function in Ptr before Tsc2 loses the 21 nt?

Response: ToxB and ToxB-like genes are old and fairly widespread. A recent study by Hafez et al. (2024; MPMI) revealed 20 different haplotypes among ToxB genes in P. tritici-repentis. They further showed that ToxB-like genes existed in 19 additional related species. It is thought that P. tritici-repentis acquired ToxB vertically, but there may have been a horizontal transfer event among species at some point. The ToxB1 haplotype is the most prevalent of the chlorosis-causing haplotypes, and it causes the most severe haplotype.

Also, there is evidence that ToxB genes do have an alternate function. Aboukhaddour et al. (2012) indicated that ToxB may play a role in the pathogen's ability to form appressoria. This sort of finding is becoming common-themed when we compare to other NE-wheat gene pairs in another similar system that we work on (i.e., wheat-Parastagonospora nodorum). In that system, we have cloned several wheat genes, and our collaborator has cloned multiple pathogen effector genes. In each case, they have demonstrated that the pathogen effector genes have an alternate function that contributes to the fitness of the pathogen, and the ability to target/hijack the host gene seems to have been acquired subsequently.

In the case of the Tsc2-ToxB interaction, it appears that both tsc2 and toxb alleles are quite old, and derived from relatives or ancestors. But the question as to which occurred first – the 21 nt deletion in the Tsc2 allele or the ToxB1 haplotype is still debatable, and more data is needed to address that. Our hypothesis is that the 21-nt deletion in Tsc2 was a random event that occurred first, and the mutation that resulted in the formation of the ToxB1 haplotype in the fungus occurred after, but giving that haplotype a selective advantage allowing it to proliferate and ultimately giving rise to a new race (race 5).

Reviewer #3 (Remarks to the Author):

I will recommend acceptance as is, although I listed minor suggestion. This is very impactful and through piece of work, and sure will have significant impact on our knowledge and screening for resistance to the pathogen. It is very comprehensive approach to answer a 40 years old standing question, what is the gene coding for Tsc2? not only it answer to that, but it also open whole new area in the interaction mechanisms used in plant-pathogen interactions, expanding on known classes of sensitivity genes.

added to the scientific advancement, there is a huge impact to be seen on the agriculture industry/scientists by identifying Tsc2/Exo70 as the susceptibility gene for a major necrotrophic effector in a major fungal disease of wheat. The work was extensive and is a classical example of work that one can teach in grad student courses these days. the authors went into all steps from purification of the ToxB effector to screening mapping population , followed by genetic linkage analysis, then fine mapping to narrow down the region from 5.6 cM to reach 0.1cM, enabled by screening with markers, phenotyping thousands of lines. followed by validation and forward genetic screen EMS-treated M2 families. followed by gain of function, transgenic complementation to confirm the role of Exo70 with no doubt and detailed the role 21nt indel in its function, defining several haplotypes so far. sure this work likely took several years of concentrated efforts!

I have read this manuscript over and over multiple times, but I only have very minor comments here and the authors can feel free to take it or ignore it. in the introduction, I think we have now agreed on the revised nomination of these toxins in Ptr, so page4, line 59, maybe just use ToxA, ToxB instead of Ptr ToxA and Ptr ToxB. The corresponding author was on the revised nomenclature work released few years ago as per the link https://doi.org/10.1094/PHYTO-01-23-0017-SC . And the other minor comments in the discussion on page 17 line 363, while the authors speak of 4-9% of sensitivity to ToxB in winter wheat, however in other work screening winter wheat lines from Europe showed 20% susceptibility to R5 (ToxB) isolate, while in Canadian durum wheat that percentage was about 6-7%, however in durum wheat it was over 30%. find the info in this reference DOI: 10.1080/07060661.2020.1870002 .the images are of great quality showing all the details of the work!

Response: Thank you for the suggestion to remove ‘Ptr’ from the ‘Ptr ToxB’ designation. We agree to this change because it reduces the word count, holds with the current agreements of the tan spot community, and helps to streamline the text.

Regarding the second suggestion, we thank the reviewer for mentioning the reference. We have cited the reference (no. 55) and added mention in the discussion (lines 408-409) to indicate the prevalence of ToxB sensitivity as high as 30% in durums and 20% in winter wheat lines in some regions of the world, which elevates the impact of our statement.

Reviewer #4 (Remarks to the Author):

This manuscript by Singh et al., reports the cloning and functional validation of wheat Tsc2 gene, the wheat tan spot susceptibility gene, which encodes a Exo70FX15. The

authors conducted genetic mapping, fine mapping, mutagenesis, transgenic complementation, and allelic diversity analysis to convincingly identify Tsc2 and demonstrate that its elimination through reading or gene editing results in enhanced resistance. It is interesting that a 21-nt deletion that spread through breeding provides important evolutionary and agronomic context. The study is clearly written, logically organized, and outlines and results presented from gene discovery to application.

The strength is the authors present Compelling genetic evidence based on positional cloning strategy that is rigorous, and complementation data strongly support gene identity. The genetic framework is the strongest aspect of the study. In addition, the breeding relevance is strong and relevant for crop improvement. Demonstrating marker-assisted breeding and gene editing as viable routes to improved resistance significantly enhances impact. The identification and historical tracing of the 21-nt deletion add depth and highlight unintended consequences of breeding.

The big weakness I observe includes very Limited mechanistic depth. While gene identification is convincing, the biochemical and mechanistic characterization remains relatively shallow. The conclusion that Tsc2 does not interact directly with the effector is based largely on Y2H assays, which are not definitive. In planta interaction assays such as co-IP would strengthen this claim.

Regardless, this is a strong genetic study with clear application and solid data supporting Tsc2 identification. However, the mechanistic advance is modest relative to the strength of the genetic findings. Strengthening interaction assays or expanding the mechanistic framework would elevate the manuscript's impact.
The work is publishable and valuable, but additional mechanistic clarity would significantly enhance its contribution.

Response: Thank you for your time in reading and reviewing the manuscript, and we sincerely appreciate your feedback. We agree that the work is heavily weighted in genetics, which we used to identify and characterize the Tsc2 gene as thoroughly as possible. Regarding your comment related to the limitations of Y2H assays, we also agree. However, given that ToxB is known to remain in the apoplast and our finding that Tsc2 remains inside the cell, we fully expected a negative Y2H result and did not feel it necessary to pursue more evidence for the lack of a direct interaction. The next phase of research to characterize this interaction will likely focus on trying to identify proteins that interact with ToxB and proteins that interact with Tsc2 to begin fitting together the mechanisms involved in the pathway that leads to a compatible interaction. But we feel that is beyond the scope of the present research and will make for a fascinating Chapter 2!

Appendix related to addressing Comment 6 from Reviewer 1:

Response: We thank the reviewer for this thoughtful and constructive suggestion. To address it, we performed an additional analysis of the geographic and regional distribution of *Tsc2* haplotype classes using Supplementary Table 16, which contains the Global Durum Panel country or breeding-program/development labels, *Tsc2* presence/absence calls, H1/H3 haplotype calls, Ptr ToxB phenotype classes, and quantitative reaction scores.

The goal of this analysis was to determine whether the H1 susceptible haplotype is geographically clustered, whether the insensitive H3 haplotype or *Tsc2*-absent/null class shows a different distribution, and whether the available data support links to breeding history or agricultural practices. We therefore summarized the data at the country/development-label level, grouped countries into broader geographic regions to reduce country-level sparsity, and evaluated the degree of sampling imbalance.

Figure R1 shows the country-level distribution of H1, H3, and Null/NA classes. In the pie-chart map, each country label is represented by a pie chart, with red indicating H1, blue indicating H3, and gray indicating Null/NA; pie size is proportional to the number of lines. The companion H1-frequency map shows H1 frequency among all lines, with point color indicating H1 frequency and point size indicating sample size. This figure directly addresses the reviewer's request for a geographic distribution summary and shows that H1 is present across multiple sampled countries rather than being restricted to one geographic location. Because many individual country labels were represented by small sample sizes, we also grouped countries into broader geographic regions and retained breeding program/development labels as a separate category (Figure R2). These broader summaries show compositional heterogeneity across the panel, but tests that include all lines, including Null/NA lines, therefore reflect both *Tsc2* presence/absence and H1/H3 distribution. For this reason, these tests are useful for describing panel structure but should not be interpreted as evidence for the geographic origin of H1.

We therefore performed a more targeted analysis focused on H1 versus H3 among *Tsc2*-positive country-label lines (Figure R3). This comparison is the most relevant test for asking whether the susceptible H1 haplotype differs geographically from the insensitive H3 haplotype. The broad-region H1-versus-H3 comparison was not significant at $P < 0.05$ by Monte Carlo conditional testing, $P = 0.066$, and the collapsed-region sensitivity analysis gave $P = 0.526$. These results suggest that the current panel does not provide strong statistical support for regional enrichment of H1 after accounting for sparsity. We also quantified the representation of country/development labels (Figure R4). This analysis showed that sampling is highly uneven: a few labels contribute a large proportion of the panel, whereas many labels contain only a small number of accessions. This imbalance limits the strength of any geographic interpretation and makes it difficult to connect the observed haplotype distribution to regional breeding history or specific agricultural practices such as conservation tillage.

As a quality-control step, we confirmed that the haplotype classes used in the geographic analysis are concordant with Ptr ToxB phenotype (Figure R5). H1 lines were sensitive, whereas H3 and Null/NA lines were insensitive, and quantitative reaction scores supported this classification. We also evaluated accession status (Figure R6). H1 versus non-H1 differed between landrace and modern lines when all lines were included, but H1 versus H3 did not differ among *Tsc2*-positive lines. This suggests that the all-line accession-status signal is driven mainly by *Tsc2* presence/absence rather than by a robust difference in the H1/H3 ratio.

Overall, with this analysis, we followed the reviewer's recommendation thoroughly by generating geographic maps, broader regional summaries, uncertainty estimates, sampling-imbalance summaries, phenotype-validation analyses, and accession-status comparisons. The data support the addition of descriptive geographic figures and charts. However, because sample sizes are uneven across countries/development labels and because some labels correspond to breeding programs or development sources rather than precise collection sites, we do not believe the current dataset supports a definitive conclusion about the geographic origin of H1 or a direct association with specific agricultural practices. A larger and more geographically balanced panel would be required to draw such conclusions.

References in response:

1. Hafez, M. *et al.* Evolution of the ToxB gene in *Pyrenophora tritici-repentis* and related species. *Mol. Plant-Microbe Interact.* **37**, 327–337 (2024).
2. Aboukhaddour, R. *et al.* RNA-mediated gene silencing of ToxB in *Pyrenophora tritici-repentis*. *Mol. Plant Pathol.* **13**, 318–326 (2012).
3. Holden, S. *et al.* A lineage-specific Exo70 is required for receptor kinase–mediated immunity in barley. *Sci. Adv.* **8**, eabn7258 (2022).

Figures to address the reviewer's comment

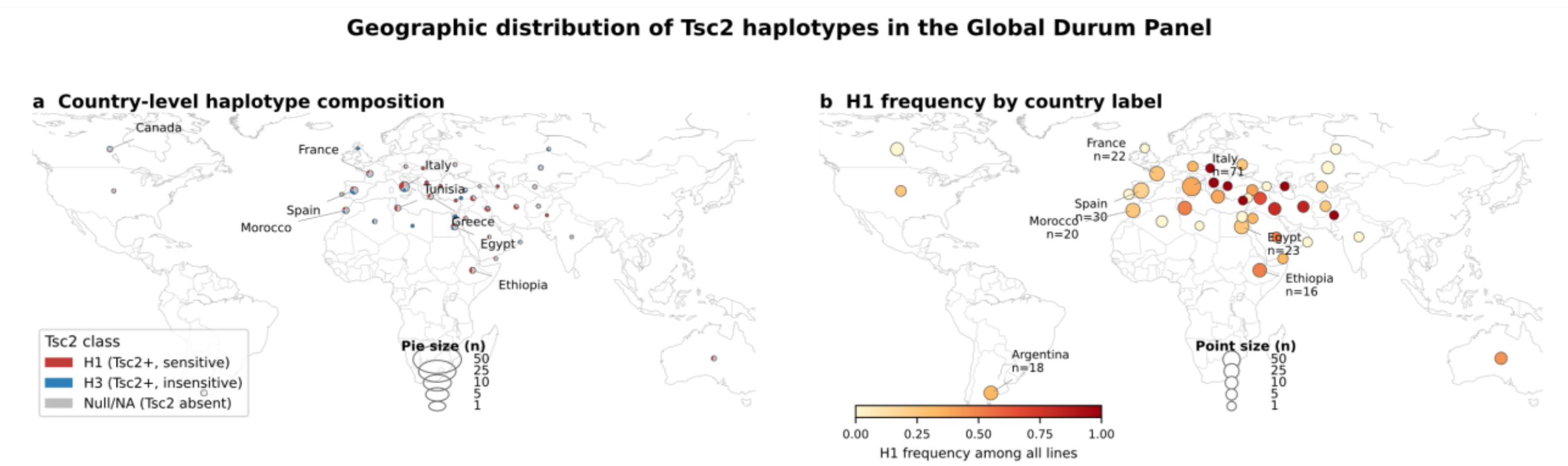

Figure R1. Country-level distribution of *Tsc2* haplotype classes in the Global Durum Panel. The analysis used Supplementary Table 16 to address whether the H1 susceptible haplotype is geographically clustered relative to H3 and *Tsc2*-absent/null lines. In panel **a**, pie charts summarize the proportions of H1, H3, and Null/NA for each country label, with pie size proportional to the number of lines. In panel **b**, points show H1 frequency among all lines, with color indicating H1 frequency and point size indicating sample size. ICARDA, CIMMYT, and DesertDurum were treated as breeding program/development labels and summarized separately rather than assigned to country coordinates. The figure shows that H1 occurs across multiple sampled countries, but country representation is uneven, limiting conclusions about H1 geographic origin.

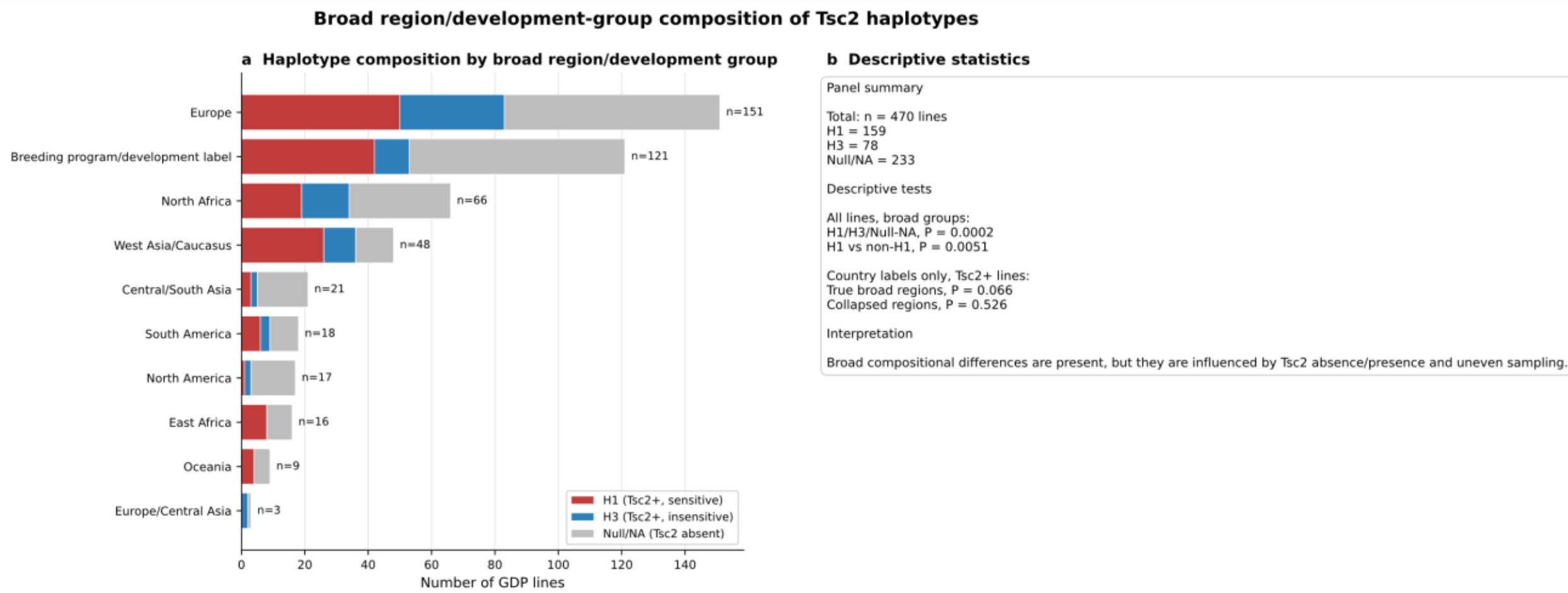

Figure R2. Broad region/development-group composition of *Tsc2* haplotypes. Countries were grouped into broader regions to reduce sparsity in Supplementary Table 16, while breeding program/development labels were retained as a separate category. Panel **a** shows the number of GDP lines classified as H1, H3, or Null/NA in each broad region/development group. Panel **b** summarizes descriptive association tests. Tests using all lines show compositional heterogeneity, but these tests include *Tsc2*-absent/null lines and therefore reflect both *Tsc2* presence/absence and H1/H3 distribution. The more targeted comparison of H1 versus H3 among Tsc2-positive country-label lines does not provide strong evidence for regional enrichment, and collapsed-region sensitivity analysis further supports a cautious interpretation.

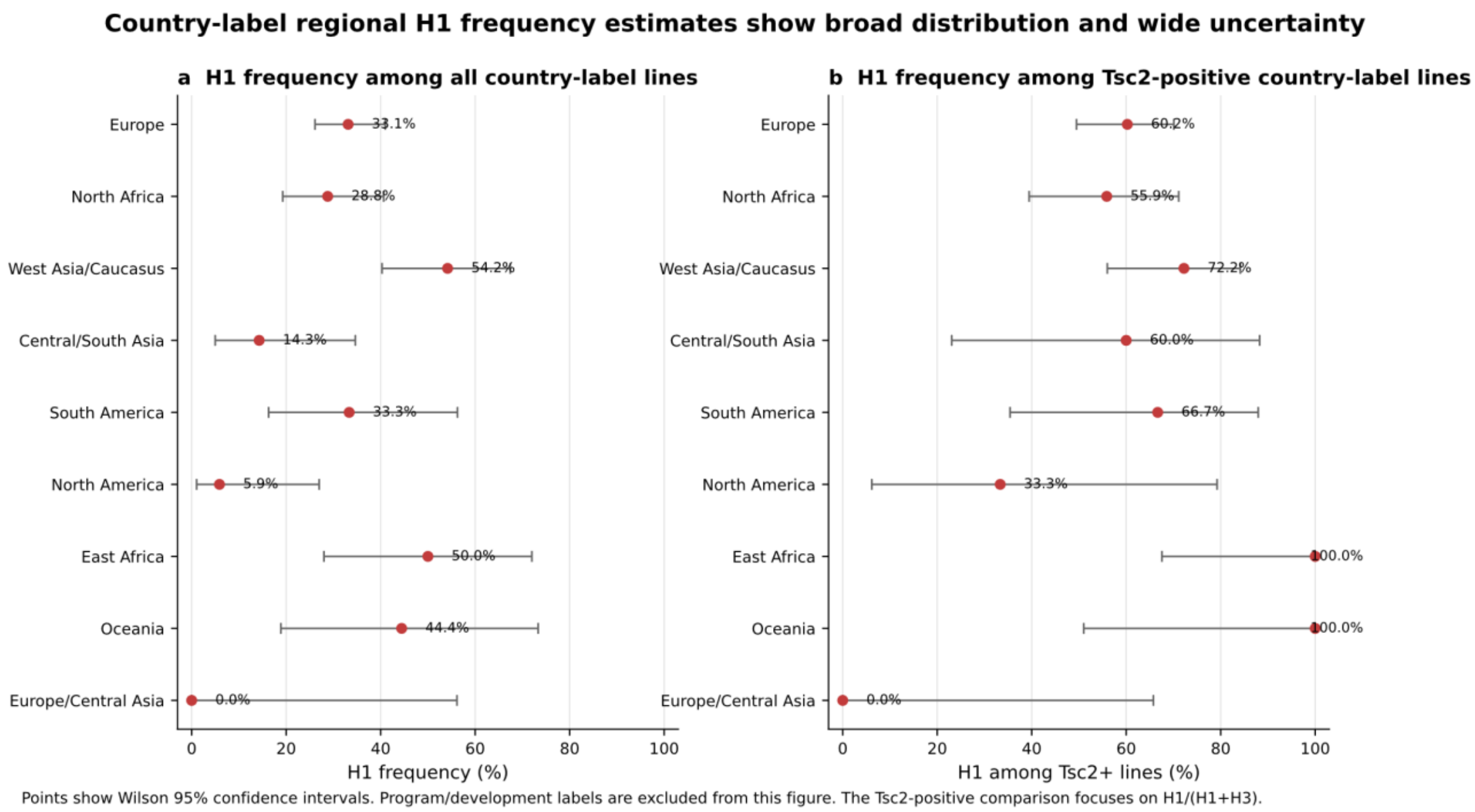

Figure R3. Regional H1 frequency estimates and uncertainty. Using Supplementary Table 16, breeding program/development labels were excluded so that the analysis focused on country-label lines only. Panel **a** estimates H1 frequency among all lines from each broad geographic region, whereas panel **b** estimates H1 frequency only among *Tsc2*-positive lines as H1/(H1+H3). Wilson 95% confidence intervals show the uncertainty around each estimate. This figure was included to make the limitation transparent: H1 appears in multiple regions, but low sample sizes in several regions produce wide confidence intervals, so apparent frequency differences should not be overinterpreted.

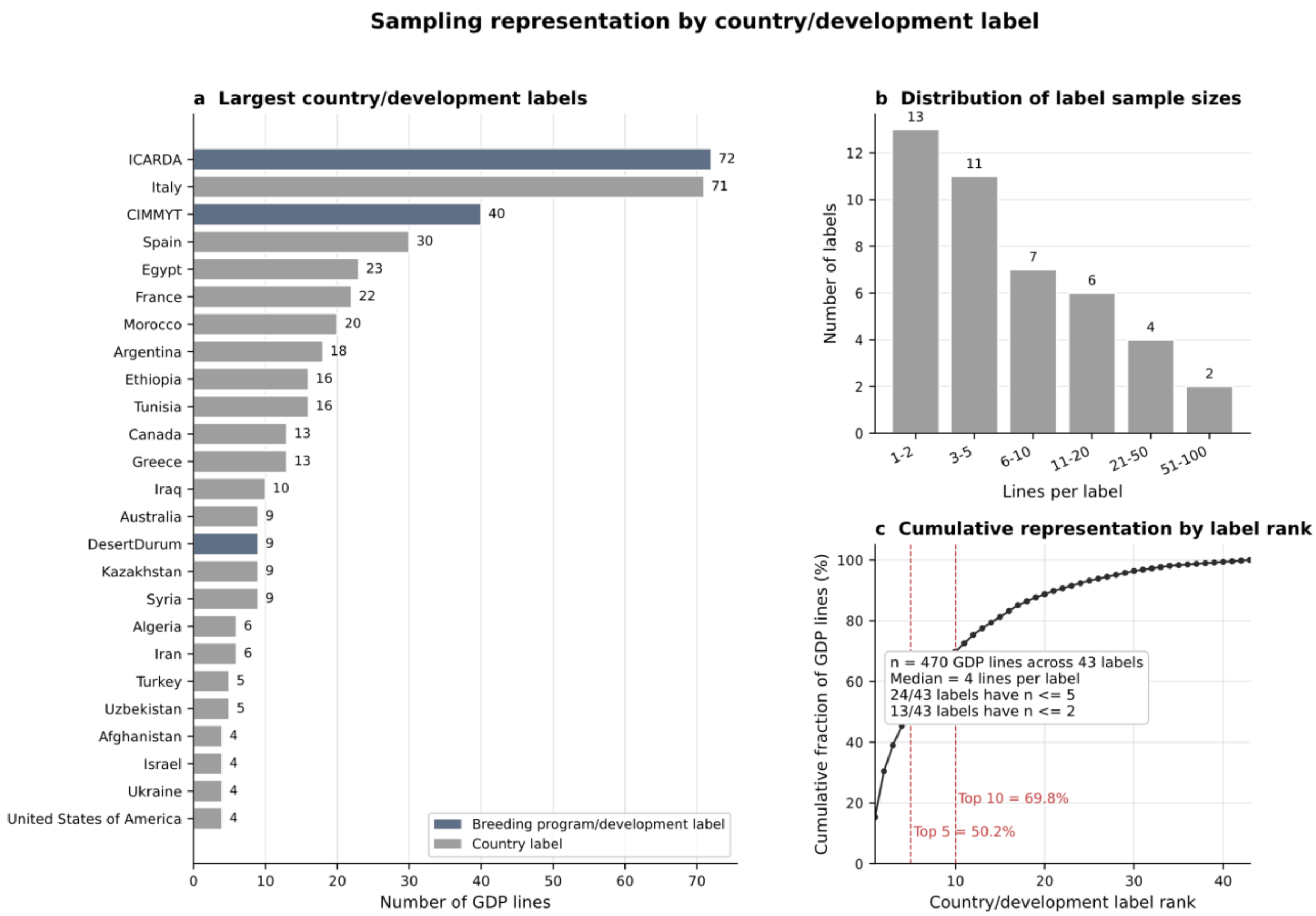

Figure R4. Sampling representation across country/development labels in the Global Durum Panel. The analysis used Supplementary Table 16 to evaluate whether the dataset was balanced enough for robust geographic inference. Panel **a** shows the largest country/development labels and highlights breeding program/development labels. Panel **b** shows the distribution of sample sizes per label, and panel **c** shows cumulative representation by ranked label. The figure demonstrates that the GDP is not evenly sampled across countries or development labels; a small number of labels contribute a large proportion of the panel, whereas many labels have few lines. This sampling structure is a major reason that the geographic analysis should be presented descriptively.

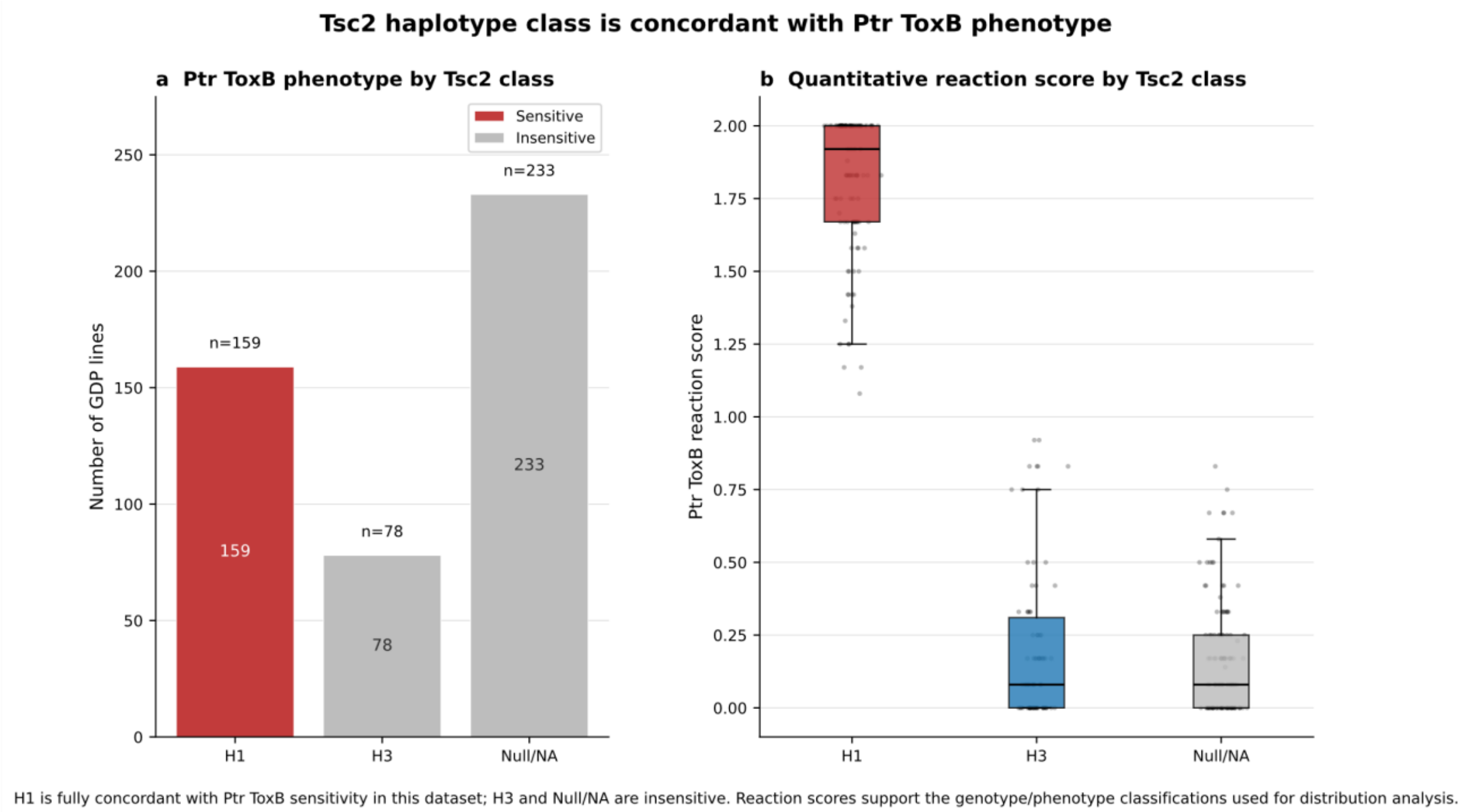

Figure R5. Concordance between *Tsc2* haplotype class and Ptr ToxB phenotype. Supplementary Table 16 contains both *Tsc2* class information and Ptr ToxB reaction data, allowing the haplotype classes used in the geographic analysis to be checked against phenotype. Panel **a** shows that H1 lines are sensitive, whereas H3 and Null/NA lines are insensitive. Panel **b** shows that quantitative reaction scores separate H1 from H3 and Null/NA. This analysis supports the use of H1, H3, and Null/NA categories in the geographic and regional summaries, but it is not intended to infer geographic origin.

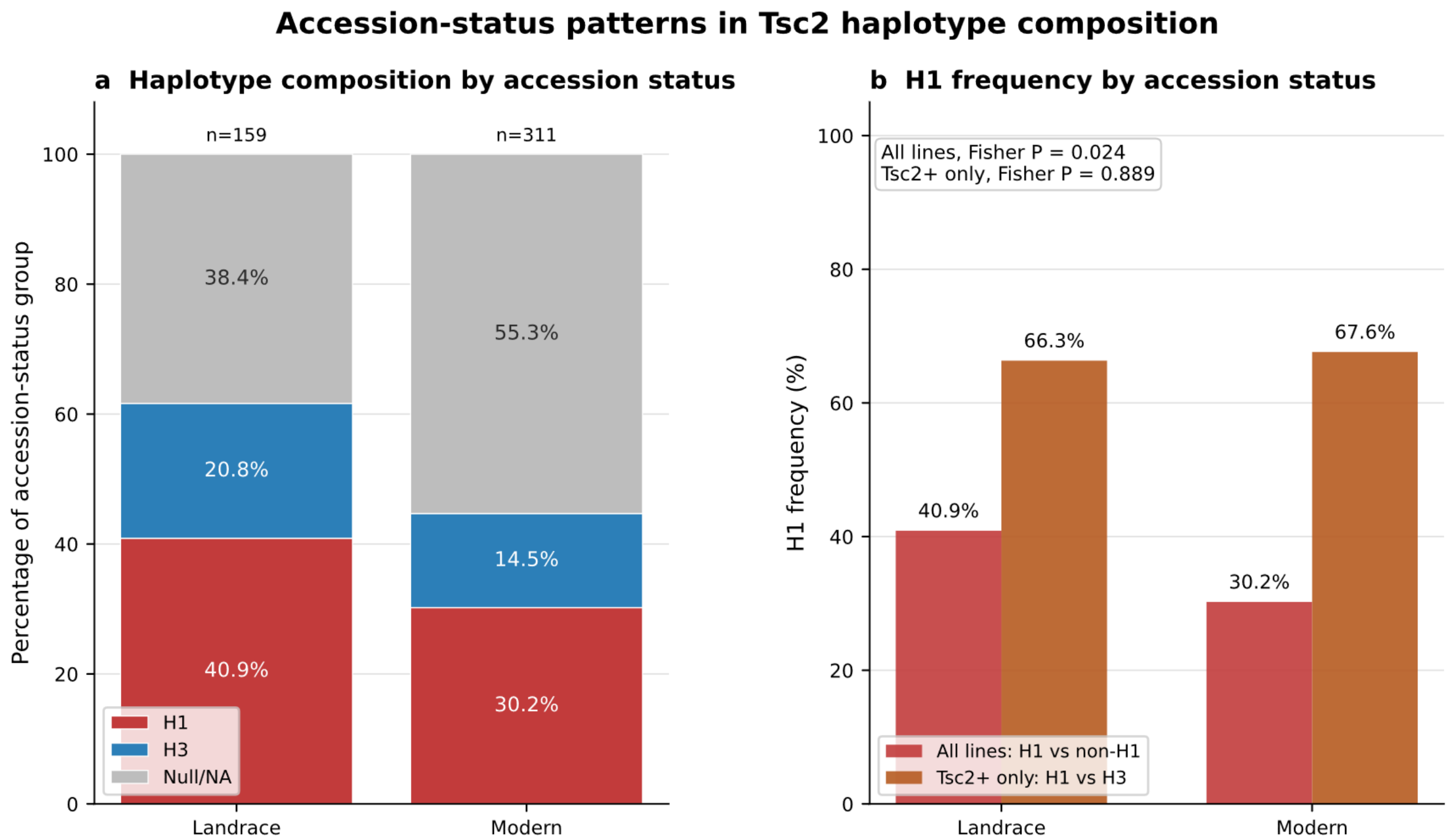

Figure R6. Accession-status patterns in *Tsc2* haplotype composition. Supplementary Table 16 was used to compare landrace and modern GDP lines. Panel **a** shows the proportional composition of H1, H3, and Null/NA for each accession-status group. Panel **b** separates the all-line H1-versus-non-H1 comparison from the *Tsc2*-positive H1-versus-H3 comparison. The all-line comparison suggests a difference by accession status, but the *Tsc2*-positive comparison does not. Therefore, this analysis supports a cautious interpretation: accession status may influence overall Tsc2 presence/absence patterns, but the current dataset does not show a robust H1-versus-H3 enrichment by accession status.
